# Quantitative Trait Loci Mapping and Candidate Gene Analysis of Low Temperature Tolerance in Cucumber Seedlings

**DOI:** 10.3389/fpls.2019.01620

**Published:** 2019-12-11

**Authors:** Shaoyun Dong, Weiping Wang, Kailiang Bo, Han Miao, Zichao Song, Shuang Wei, Shengping Zhang, Xingfang Gu

**Affiliations:** Institute of Vegetables and Flowers, Chinese Academy of Agricultural Sciences, Beijing, China

**Keywords:** cucumber, low temperature tolerance, quantitative trait locus mapping, *in silico* bulked segregant analysis, candidate gene analysis

## Abstract

Cucumber (*Cucumis sativus* L.) is an economically important vegetable crop worldwide, but it is sensitive to low temperatures. Cucumber seedlings exposed to long-term low temperature stress (LT), i.e., below 20°C during the day, and 8°C at night, exhibit leaf yellowing, accelerated senescence, and reduced yield, therefore posing a threat to cucumber production. Studying the underlying mechanisms involved in LT tolerance in cucumber seedlings, and developing germplasm with improved LT-tolerance could provide fundamental solutions to the problem. In this study, an F_2_ population was generated from two parental lines, “CG104” (LT-tolerant inbred line) and “CG37” (LT-sensitive inbred line), to identify loci that are responsible for LT tolerance in cucumber seedlings. Replicated phenotypic analysis of the F_2_-derived F_3_ family using a low-temperature injury index (LTII) suggested that the LT tolerance of cucumber seedlings is controlled by multiple genes. A genetic map of 990.8 cM was constructed, with an average interval between markers of 5.2 cM. One quantitative trait loci (QTL) named *qLTT5.1* on chromosome 5, and two QTLs named *qLTT6.1* and *qLTT6.2* on chromosome 6 were detected. Among them, *qLTT6.2* accounted for 26.8 and 24.1% of the phenotypic variation in two different experiments. Single-nucleotide polymorphism (SNP) variations within the region of *qLTT6.2* were analyzed using two contrasting *in silico* bulks generated from the cucumber core germplasm. Result showed that 214 SNPs were distributed within the 42-kb interval, containing three candidate genes. Real-time quantitative reverse transcription PCR and sequence analysis suggested that two genes *Csa6G445210*, an auxin response factor, and *Csa6G445230*, an ethylene-responsive transmembrane protein, might be candidate genes responsible for LT tolerance in cucumber seedlings. This study furthers the understanding of the molecular mechanism underlying LT tolerance in cucumber seedlings, and provides new markers for molecular breeding.

## Introduction

Cucumber (*Cucumis sativus* L.) is an economically important vegetable crop. Global production reached 80 million metric tons in 2016, with a steady increase in production since 1998 (Chen et al., 2019). Cucumber originated from tropical regions, and the suitable temperature range for growth is 18–30°C. As an annual vegetable crop which is sensitive to low temperature (LT) (Cabrera et al., 1992), cucumber easily suffers from LT injury in winter and early spring in regions such as Northern China, necessitating greenhouse-cultivation in these regions. Depending on its intensity and duration, LT-stress may be divided into long-term moderate LT (below 20°C during daytime and 8°C at night for days or weeks) and short-term extreme LT (15°C during the day and 4°C at night for hours) (Smeets and Wehner, 1997). Long-term moderate LT stress occurs more often during production, leading to yellowed leaves, accelerated senescence, and decreased yield (Smeets and Wehner, 1997), such that even this moderate stress has become a serious obstacle for the cucumber production industry. Thus, LT tolerance at the seedling stage is a desirable trait for cucumber breeding. With the continuous expansion of the cucumber planting area into early spring and winter in China, it is critical to identify candidate genes responsible for LT tolerance, and to develop resistant cultivars.

LT tolerance of cucumber seedling is a complex trait controlled by multiple alleles at multiple loci (Chung et al., 2003; Kozik and Wehner, 2008; Kozik et al., 2012). Chung et al. (2003) found that LT tolerance at the seedling stage is maternally inherited, and that the gene conferring resistance comes from the chloroplast genome of the female parent “Chipper.” Kozik and Wehner (2008) reported that the LT tolerance of cucumber seedlings was controlled by a single dominant gene *Ch*. More recently, a study of two genotypes, LT-tolerant “PI390953” and cold-sensitive “Gy14” suggested that LT tolerance of cucumber seedlings was determined by two genes (Kozik et al., 2012).

There are few recent reports on the gene or quantitative trait loci (QTL) mapping of LT tolerance in cucumber seedlings. A few studies were carried out under short-term extreme LT (Li, 2014; Wang, 2014; Zhou, 2015). When a qualitative chilling injury index (CII) was used as an indicator of LT stress, one simple sequence repeat (SSR) marker closely linked to a LT-tolerant gene on chromosome 6 was identified (Li, 2014). Zhou (2015) identified six loci associated with LT tolerance on chromosome 3. Wang (2014) used CII and a recovery index as indicators of LT tolerance, and identified three QTLs on chromosome 3 and one QTL on chromosome 7, respectively. However, there are no reports of LT-tolerant QTLs or gene mapping using long-term moderate LT as a selective criterion, and long-term moderate LT stress occurs more often during cucumber production.

Since the International Cucumber Genome Team announced the whole genome sequence of “Chinese long” line 9930 (Huang et al., 2009), multiple lines have been re-sequenced, revealing all genotypic variability (Qi et al., 2013). A new approach called “*in silico* BSA (bulked segregant analysis)” was reported (Bello et al., 2014). Its application avoids the construction of near isogenic lines and greatly improves the efficiency of gene mapping. The method is based on the results of gene/QTL mapping and genetically diverse germplasm with known phenotype. Two bulks with contrasting target phenotype are constructed, and the single-nucleotide polymorphism (SNP) variations within the target QTL region between two bulks are inspected. The SNPs that are identical within each bulk but different between two bulks may be highly associated with the target phenotype. With this method, Li et al. (2016) quickly narrowed the *Cn* locus into a 16-kb region, and Bo et al. (2019) fine-mapped the fruit spine density related major QTL *qfsd6.2* to a 50-kb region, and finally identified the candidate gene *Csgl3*.

So far, studies on LT tolerance in cucumber mainly focused on the LT stress conditions applied, genetic mechanisms and germplasm evaluation criteria (Kozik and Wehner, 2008; Kozik et al., 2012). However, very few QTL analyses of LT tolerance in cucumber have been reported and no genes have been identified. Such information would provide practical tools to enhance breeding strategies for this trait. Therefore, the objective of this study was to identify QTL and candidate genes responsible for LT stress tolerance. Our preliminary study showed that the cucumber inbred line “CG104” exhibits high tolerance to LT, while inbred line “CG37” is sensitive to LT. An F_2_ population from a cross of these two inbred lines was constructed, and used for QTL mapping. Then, SNP variations within the major QTL region between two contrasting *in silico* bulks were analyzed. Sequence analysis and quantitative reverse transcription PCR analysis were then performed to identify the potential candidate gene. This study thus helps to further the understanding of the molecular mechanism underlying LT tolerance in cucumber seedlings, and provides new tools for breeding LT-tolerant cucumber germplasm *via* molecular marker-assisted breeding.

## Materials and Methods

### Plant Materials

Two cucumber inbred lines, “CG104” (LT-tolerant) and “CG37” (LT-sensitive) were used in this study. “CG37” and “CG104” were crossed to generate an F_1_ (“CG104” x “CG37”) and F_1_' (“CG37” x “CG104”) populations. The F_1_ (“CG104” x “CG37”) was self-pollinated to produce an F_2_ population of 189 individuals. A total of 189 F_2:3_ families were derived from F_2_ populations accordingly. Moreover, 10 core germplasms with high LT-sensitive or LT-resistant phenotype (**Table S1**) that have been resequenced (Qi et al., 2013) were used for *in silico* BSA analysis. The F_2_ population was used to verify mutation sites in candidate genes. All materials were preserved by the Cucumber Research Group, Institute of Vegetables and Flowers (IVF), Chinese Academy of Agricultural Sciences (CAAS).

### Investigation of Low Temperature Injury at Seedling Stage

**Figure 1:**
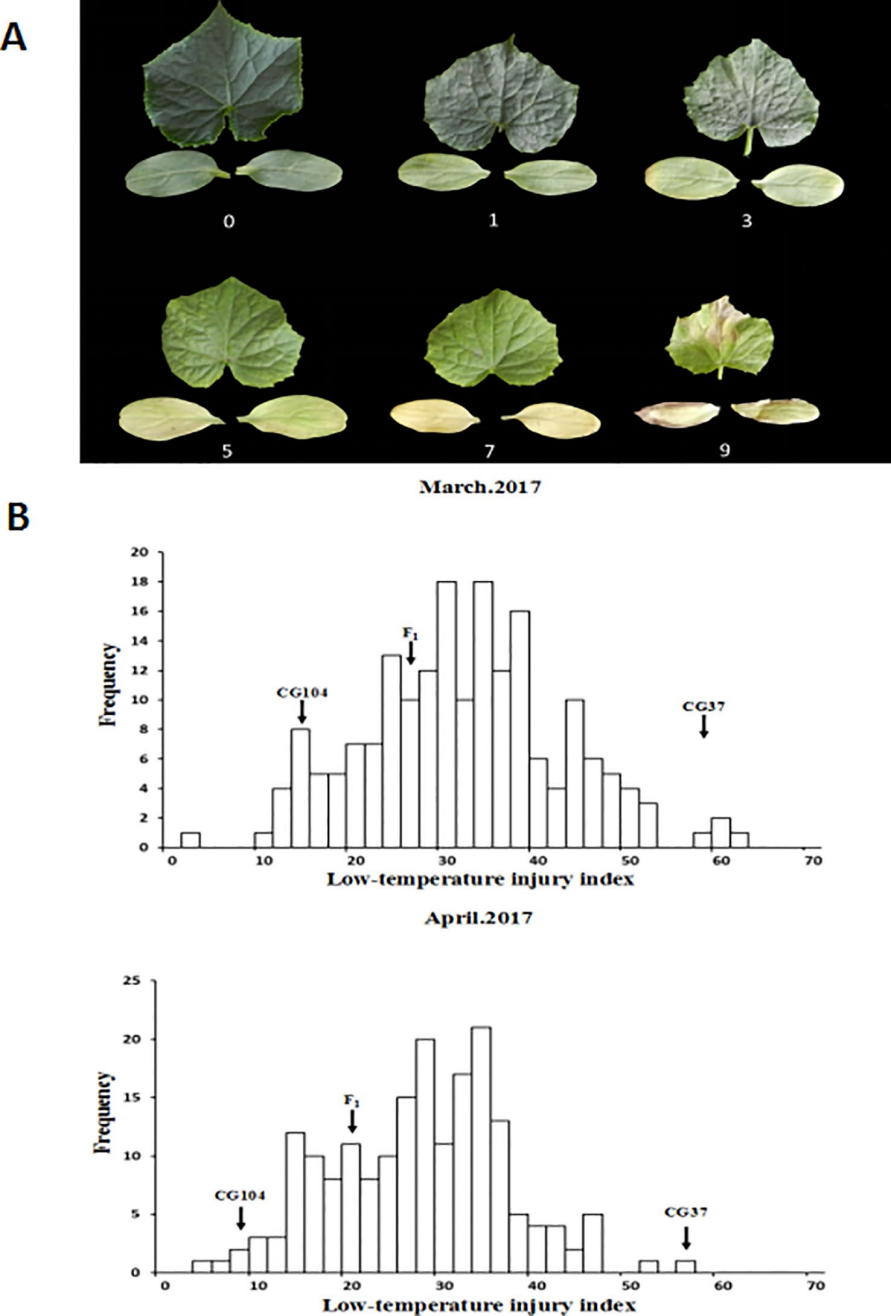
Phenotypic characterization of low temperature tolerance in F_2:3_ populations. **(A)** Scales of low-temperature injury index (LTII) based on the degree of yellowing and dryness of the cotyledons and the first true leaf. 0: no symptoms on either the cotyledons or first true leaf; 1: cotyledons were slightly yellow, while the first true leaf was still green; 3: cotyledons were yellow-green with yellowed edges, while the first true leaf was light green; 5: cotyledons were yellowed in large scale, while the first true leaf was yellow-green; 7: cotyledons were completely yellowed, while the first true leaf was yellow-green; 9: cotyledons and the first true leaf dried. **(B)** Frequency distribution of LTII in F_2_ populations characterized in March 2017 and April 2017.

LT treatments were carried out twice in the greenhouse and plastic greenhouse of the IVF, CAAS at Changping, Beijing (40°13'N, 116°05'E) in early spring, respectively (**Table S2**). Three-week-old seedlings of the two parental lines, F_1_ and F_2_-derived F_3_ families were exposed to 15–17°C for 2 weeks, and the LT injury was classified into six grades, based on the degree of yellowing and dryness of the cotyledons and the first true leaf (**Figure 1**). The index used was as follows: 0: no symptoms on either the cotyledons or the first true leaf; 1: cotyledons were slightly yellow, while the first true leaf was still green; 3: cotyledons were yellow-green with yellowed edges, while the first true leaf was light green; 5: cotyledons were yellowed in large scale, while the first true leaf was yellow-green; 7: cotyledons were completely yellowed, while the first true leaf was yellow-green; 9: cotyledons and the first true leaf dried.

A low-temperature injury index (LTII) was used as an indicator to indicate the LT tolerance of each plant. The formula used for the calculation of LTII refers to Wang et al. (2019): LTII = (0×S_0_+1×S_1_+3×S_3_+5×S_5_+7×S_7_+9×S_9_)/N×9. S_0_–S_9_ indicates the number of plants corresponding to each grade. N indicates the total number of plants. Three replicates were set for each treatment, and eight plants of each replicate were investigated. The LTII in two experiments was evaluated by two people, respectively.

### Deoxyribonucleic Acid Extraction and Simple Sequence Repeats Marker Analysis

A modified CTAB (cetyltrimethylammonium ammonium bromide) procedure (Wang et al., 2006) was applied to extract genomic DNA from the second leaf of each two-leaf stage seedlings. The DNA concentration and quality was examined by electrophoresis on a 1% (w/v) agarose gel and NanoDrop ND-1000 spectrophotometer (NanoDrop Technologies, Wilmington, DE, USA). A total of 1,288 SSR markers designed based on the genome sequence of cucumber (Ren et al., 2009) were used to screen the polymorphism of the two parents. Then, a linkage map was constructed with selected polymorphic markers. PCR amplification and gel electrophoresis were conducted referring to the method of Song et al. (2016).

### Linkage Map Construction and Quantitative Trait Locus Mapping

Polymorphic primers were used for genotyping F_2_ individuals. QTL analysis was performed with the R/QTL software package (http://www.rqtl.org/). Using individual plant data, a whole genome scan was performed to map the QTLs with composite interval mapping (CIM) procedures (Weng et al., 2015). Genome-wide logarithm of the odds (LOD), threshold values (*P* < 0.05) for declaring the presence of QTLs were determined using 1,000 permutations. For each detected QTL, a 2-LOD support interval was calculated and defined by left and right markers. The QTL naming format is the abbreviation of the character (i.e., low temperature tolerance—LTT), chromosome (Chr.) number and locus number.

### Defining the Interval Containing the Major Quantitative Trait Locus Using *In Silico* Bulked Segregant Analysis

To narrow down the region harboring the major QTL, the association between SNPs and LT tolerance in core germplasm was studied using the *in silico* BSA strategy. The LT tolerance of 87 sequenced core germplasm (CG) seedlings was previously evaluated in our lab (Wang et al., 2019), two-leaf stage seedlings of CG lines were exposed to 12°C for 11 d and 19.3°C for 14 d, respectively. The LTT of each line was evaluated using the same method as that used to phenotype the F_2:3_ families described above. Five highly LT-resistant CG lines (“CG29,” “CG56,” “CG61,” “CG90,” “CG104”) and five highly LT-sensitive CG lines (“CG10,” “CG21,” “CG43,” “CG109,” “CG37”) were selected to generate LT-resistant and LT-sensitive bulks (**Table S1**, **Figure S2**). The 10 CG lines have different geographical origin, and were re-sequenced (Qi et al., 2013). The sequence data was retrieved from National Center for Biotechnology Information (NCBI) Short Read Archive (SRA; Leionen et al., 2011) under accession SRA056480 (https://www.ncbi.nlm.nih.gov/sra/?term=SRA056480). The detailed accession number of each CG line was listed in **Table S1**. Then, the SNPs located within the *qLTT6.2*. region (20,591,185 to 21,186,690 bp) were searched (**Table S3**) and imported into PilotEdit software. SNPs that were identical *within* each bulk while different *between* bulks, were considered to be associated with the LT tolerance.

### Real-Time Quantitative Reverse Transcription Polymerase Chain Reaction Analysis

The parental lines at the two-leaf stage were exposed to 14 h light at 18°C and 10 h dark at 10°C, the second true leaf of each seedling was harvested at 0, 10, 24, 48, 96, 144, 216, 288, 384, and 480 h after LT exposure, respectively, and flash frozen in liquid nitrogen. Total RNA was isolated and the first-strand complementary DNA (cDNA) was synthesized. The qPCR primers were designed with Primer Premier 6.0 (http://www.premierbiosoft.com/primerdesign/index.html). The qRT-PCR was performed using SYBR Premix Ex Taq^™^ (Tli RNaseH Plus) (Takara #RR420Q, Takara Bio, Inc. China) in Roche Diagnostics with Light Cycler 480 System, and PCR amplification was conducted following the instructions. *Actin1* (*Csa3G806800*) was used as the reference gene for the normalization of gene expression values (Wan et al., 2010). Each experiment was conducted with three biological replicates and three technical replicates. The relative expression data of candidate gene was analyzed using 2^−ΔΔCt^ method (Kenneth et al., 2001).

### Cloning and Sequence Analysis of Candidate Genes

The second true leaf of seedlings at the two-leaf stage was harvested for DNA extraction. Multiple pairs of primers were designed to amplify the full length candidate gene, and neighboring fragments have at least 100 bp overlap. Primers designed were listed in **Table S4**. PCR amplification was performed in a 25 µl reaction: 2.5 µl of 10X PCR buffer, 1 µl of 25X Mg^2+^, 1 µl of 10X nucleoside triphosphate, 1 µl of 10 µM forward and reverse primer, 0.25 µl of KOD FX polymerase (Toyobo, Osaka, Japan) and 250–500 ng of DNA. The PCR conditions were as follows: 94°C for 5 min, 30 cycles of (94°C for 40 s, 55°C for 50 s, 72°C for 90 s), 72°C for 10 min, and a final incubation at 4°C for use. The PCR products were then sequenced by Sangon Biotech (Beijing, China). The sequence of the candidate genes of the parental lines were analyzed using SeqMan, and the amino acid sequences were analyzed using the MEGA 6.0 software (Tamura et al., 2013). The promoter sequences of candidate genes were analyzed using the PlantCARE website (http://bioinformatics.psb.ugent.be/webtools/plantcare/html/), and the protein sequences of candidate genes were predicted by SMART software (Ponting et al., 1999).

### Phylogenetic Analysis of *Csa6G445210* and *Csa6G445230*

To further understand the relatedness of the *Csa6G445210* and *Csa6G445230* sequences, phylogenetic trees were generated. The predicted amino acid sequences encoded by *Csa6G445210* and *Csa6G445230* in cucumber and orthologues in *Cucumis melon*, *Arabidopsis thaliana*, *Brassica rapa*, *Solanum lycopersicum*, *Oryza sativa* cv. *Japonica*, and *Zea mays* were obtained using the BLASTP tool (Altschul et al., 1997) in the NCBI database. Multiple sequence alignment was implemented with ClustalW (Thompson et al., 1994) and the phylogenetic tree was structured using the MEGA 6.0 software (Tamura et al., 2013) with a neighbor-joining algorithm.

### Allelic Diversity of the Candidate Gene in F_2_ Individuals

F_2_ individuals were used to identify the correlation between the phenotype (*via* F_2:3_ families) and the SNP mutation sites at the candidate genes. Specific SNP markers at mutation sites were designed (**Table S5**) to amplify DNA from F_2_ individuals, and then the amplified products were digested for genotyping. The individuals that produce the same digestion products as “CG104,” “CG37,” and F_1_ were defined as LT resistant (a), sensitive (b), and hybrid (ab), respectively. Then, the genotype and LTII value of each F_2_ individual were compared.

### Statistical Analysis

All tests for significant differences between “CG104” and “CG37” were done using one-way ANOVA in the R environment (R core Team, 2019).

## Results

### Inheritance Analysis of Low-Temperature Tolerance in Cucumber Seedlings

To phenotype the LT tolerance of the two parental lines, and individuals in the F_1_ and F_2:3_ populations, 3-week-old seedlings were exposed to 15–17°C for 2 weeks in March 2017 and April 2017, respectively. The LTII was used to indicate the LT tolerance of each plant. Results showed that after 2 weeks of LT treatment, cotyledons and the first true leaf of “CG37” showed severe withering and yellowing, and the LTII score of “CG37” in greenhouse and plastic greenhouse were 59.1 and 57.7 respectively. However, “CG104” had no symptoms and the LTII were 14.9 and 8.7 respectively. The LTII of the F_1_ hybrids were 26.4 and 21.7, respectively, with the symptoms more inclined toward “CG104.” Frequency distribution of the LTII among F_2:3_ families obeyed a normal distribution (**Figure 1** and **Figure S1**) in two experiments (the coefficient of correlation is 0.87). An additional experiment of phenotyping F_1_ and F_1_” showed that there is no maternal effect (**Table S6**), which all together indicate that LT tolerance of cucumber at the seedling stage is a quantitative trait, controlled by multiple nuclear genes.

### Linkage Map Construction and Quantitative Trait Locus Mapping

**Figure 2:**
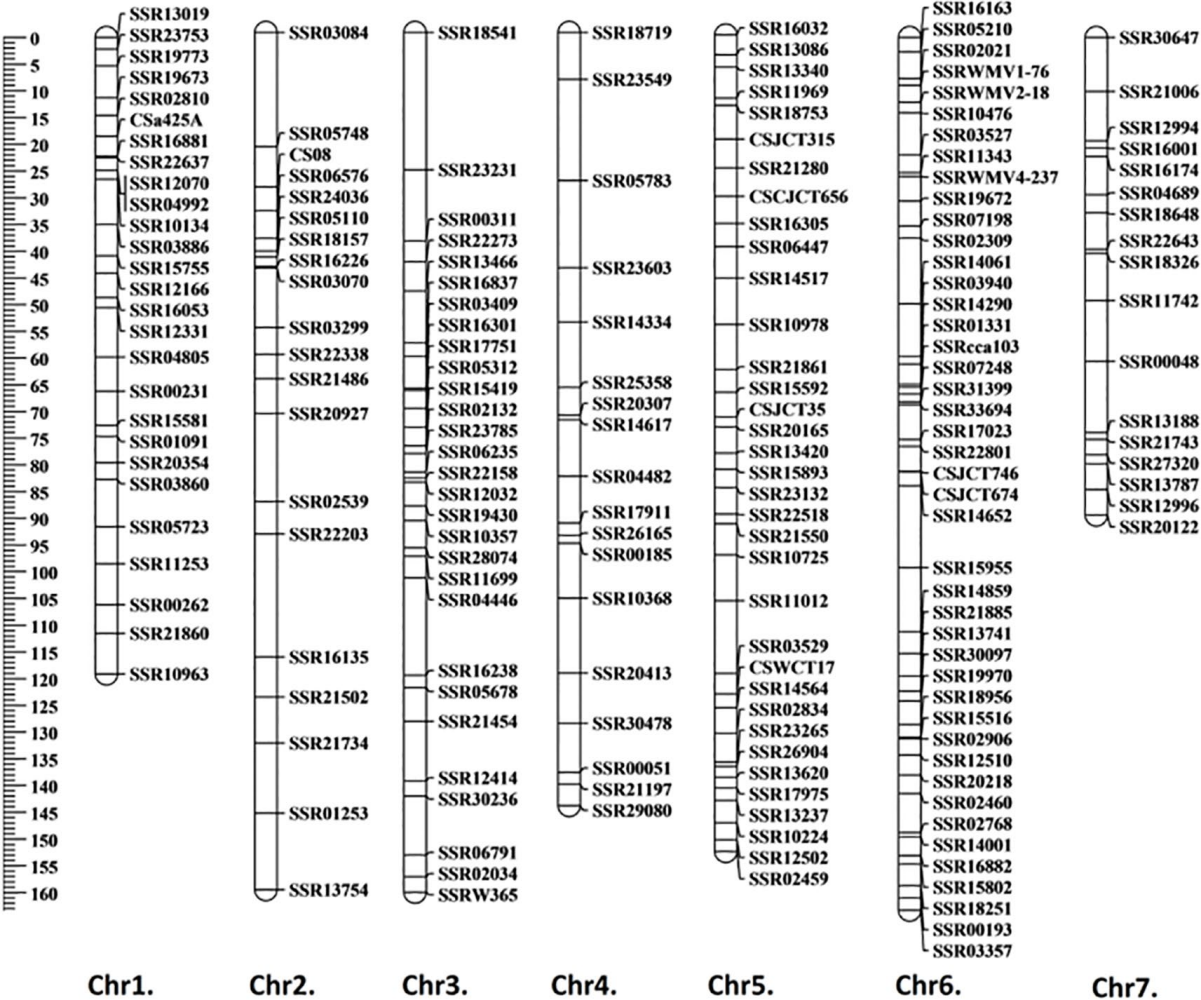
Genetic linkage map generated using F_2_ population. The generated linkage map contained 190 markers with a total length of 990.8 cM, and the average genetic distance between markers was 5.2 cM. Map distance was given in centimorgans (cM).

A total of 1,288 cucumber SSR markers were used to screen the polymorphisms between the parental lines; 509 of them showed polymorphisms, with a polymorphism rate of 39.5%. A total of 190 markers with clear electrophoresis strips were used to generate a linkage map (**Figure 2**). The markers selected were evenly distributed on seven chromosomes. The total length of the genetic map was 990.8 cM, and the average genetic distance between markers was 5.2 cM (**Table S7**). Based on the “9930” genome (Huang et al., 2009), the order of all markers on the genetic map was consistent with their physical location, therefore the map was qualified to be used for subsequent QTL mapping.

**Figure 3:**
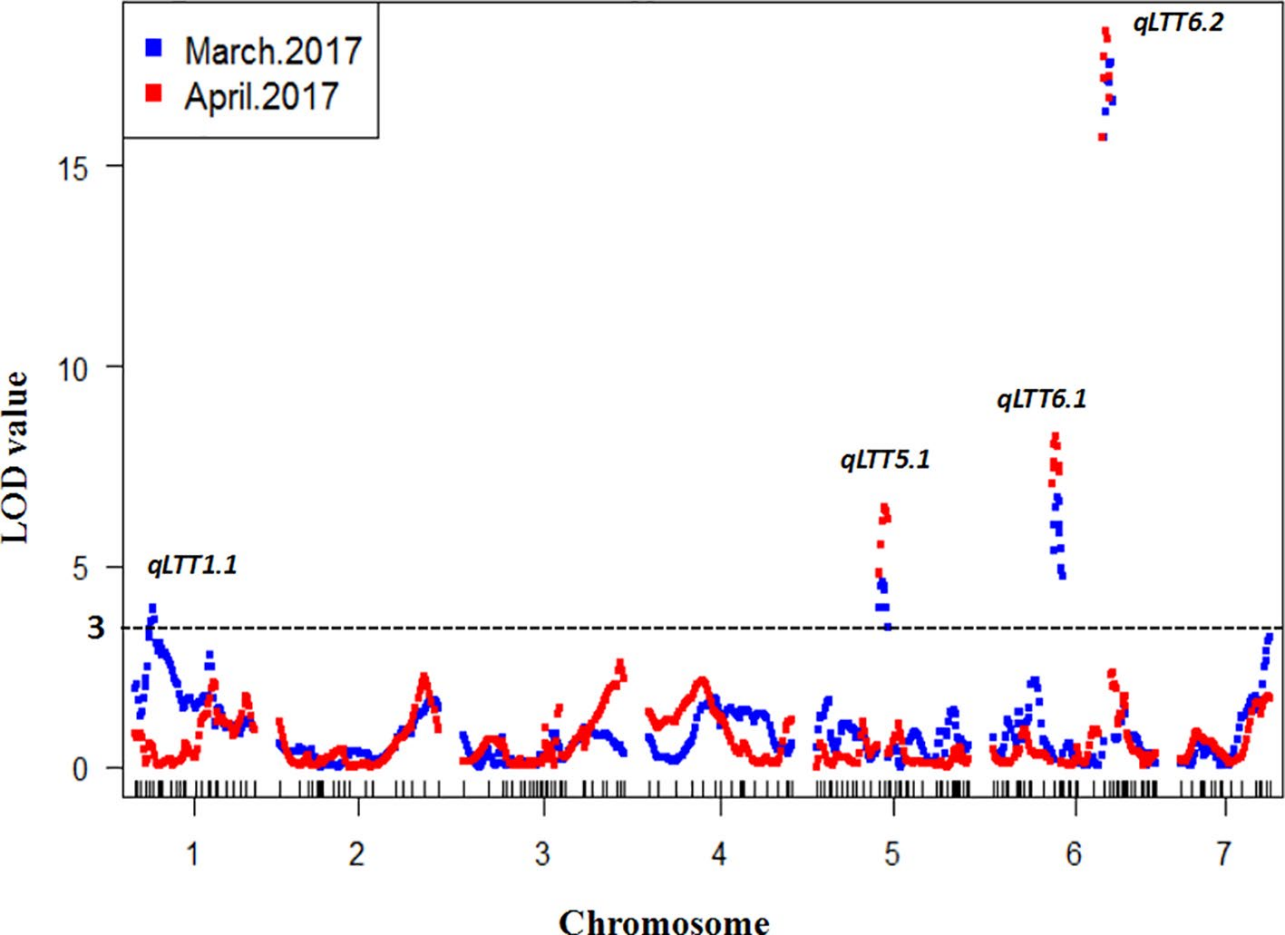
Quantitative trait locus (QTL) analysis of low temperature tolerance in cucumber seedlings. Blue squares indicates the first treatment and red square indicates the second treatment. The x-axis indicates the genetic position of each chromosome, the y-axis indicates the logarithm of the odds value. Four QTLs were detected in two experiments and three QTLs were repeatedly identified.

To detect QTL responsible for LT tolerance in cucumber seedlings, the phenotypic data for LT tolerance (LTII) from the two experiments and the genetic map constructed were used for QTL mapping. Details of each QTL detected, including map location, peak location, peak logarithm of odds (LOD) support value, confidence intervals, and percentages of total phenotypic variances explained (R^2^) were shown in **Table 1**. In total, three QTLs including *qLTT5.1* on Chr.5, *qLTT6.1* and *qLTT6.2* on Chr.6 were repeatedly detected (with a threshold of 3, and the confidence intervals of 2-LOD) in two experiments (**Table 1** and **Figure 3**). Furthermore, LOD scores of *qLTT6.2* is 18.2 and 16.8, and accounts for 26.8 and 24.1% of the phenotypic variation. Therefore, it was proposed that *qLTT6.2* is a major QTL which is responsible for LT tolerance in cucumber seedlings.

**Table 1:** Identified quantitative trait loci controlling low temperature tolerance in cucumber seedlings. LTT indicates low temperature tolerance.

Treatments	QTL	Chr.	Peak location	Peak LOD	2 LOD interval (cM)	R^2^/%
March 2017	*qLTT1.1*	1	18.5	3.6	SSR02810(14.6)–SSR16881(22.2)	8.1
	*qLTT5.1*	5	66.9	4.4	SSR15592(66.9)–CSJST35(71.5)	9.8
	*qLTT6.1*	6	63.1	6.4	SSR14290(61.1)–SSR01331(64.9)	8.0
	*qLTT6.2*	6	113.2	18.2	SSR21885(115.1)–SSR14859(119.3)	26.8
April 2017	*qLTT5.1*	5	66.9	6.0	SSR15592(66.9)–SSR20165(73.4)	13.5
	*qLTT6.1*	6	63.1	7.8	SSR14290(61.1)–SSR01331(64.9)	9.8
	*qLTT6.2*	6	113.2	16.8	SSR21885(115.1)–SSR14859(119.3)	24.1

### Reducing the Interval of Locus *qLTT6.2* Using *In Silico* Bulked Segregant Analysis

**Figure 4:**
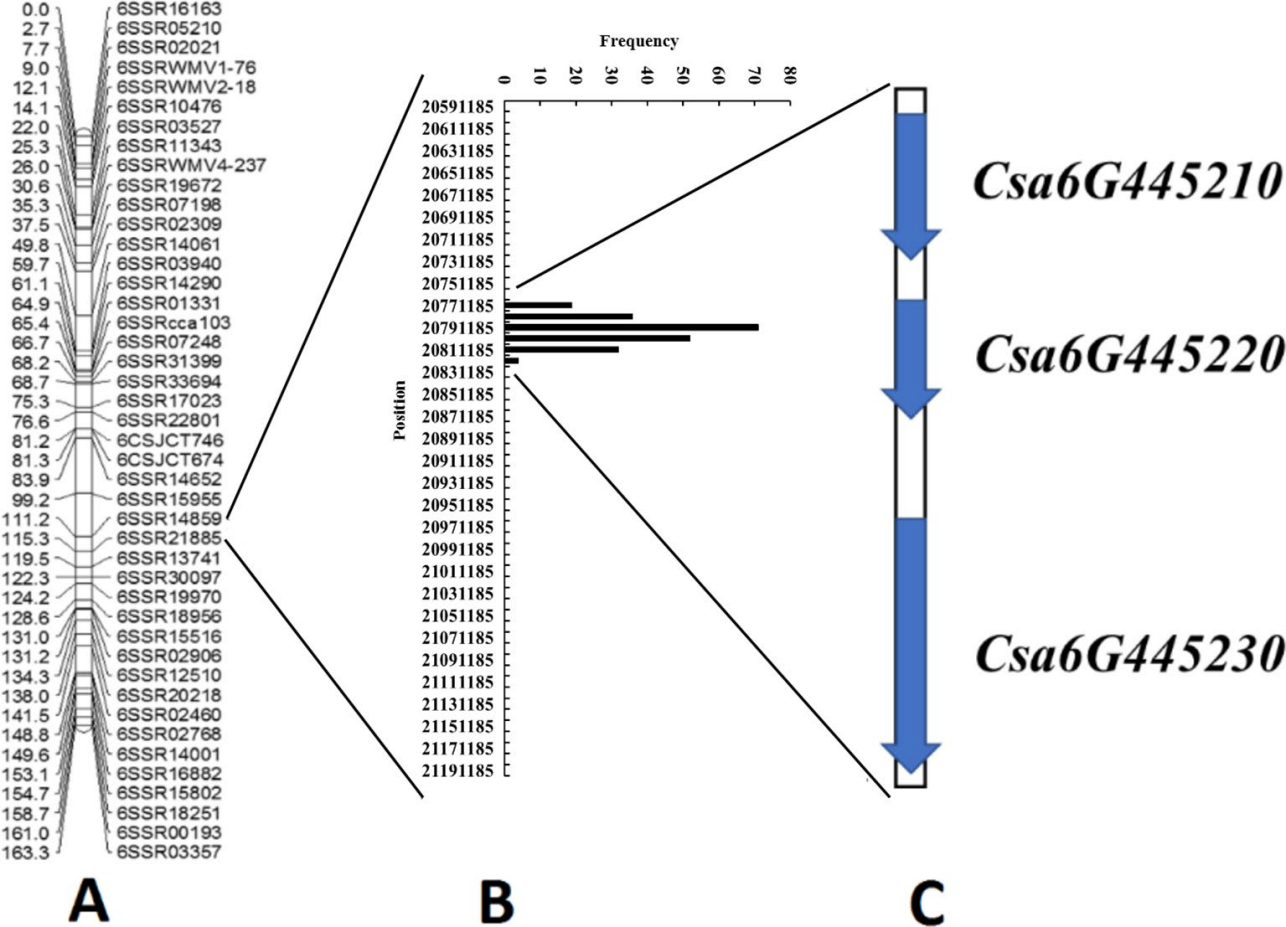
Identification of candidate genes for the *qLTT6.2* locus through combined linkage mapping and association analysis. **(A)** Linkage map for *qLTT6.2* locus on chromosome 6 based on 189 F_2:3_ families from “CG37” × “CG104.” **(B)** Distribution of 214 single-nucleotide polymorphisms in the “9930” cucumber genome, suggested that a 42-kb region was associated with the variation of low temperature tolerance in cucumber core germplasm materials. **(C)** Three candidate genes were identified in the 42-kb region.

Because *qLTT6.2* showed the highest contribution to LT tolerance in two experiments, we further conducted *in silico* BSA to narrow the region harboring this locus. Our previous study included the LT tolerance evaluation of 87 CG lines (**Figure S2**). Among these, five lines: “CG29,” “CG56,” “CG61,” “CG90,” “CG104” with strong LT resistance phenotypes, and another five lines: “CG10,” “CG21,” “CG43,” “CG109,” “CG37,” with high LT sensitive phenotypes were used to generate LT-resistant and -sensitive bulks to identify the SNPs variation. The detailed information of these 10 lines including accession name, accession number in NCBI, geographical origin, and their LT tolerance performance are listed in **Table S1**. There were 214 non-synonymous SNPs that were identical *within* each bulk, but different *between* bulks (**Table S3**), all of which were distributed within the 42-kb region (20,779,616 to 20,821,620). These data indicated that this 42 kb region was associated with LTII variation among these lines. Annotation of the 42 kb genomic region predicted three genes including one for an auxin response factor, one for a CASP-like protein, and an *A. thaliana* ethylene insensitive 2 orthologue (**Figure 4** and **Table 2**).

**Table 2:** Annotation of candidate genes responsible for low temperature tolerance.

Gene ID	Location on Chr. 6	Gene function annotation
*Csa6G445210*	20785976–20789697	Auxin response factor
*Csa6G445220*	20794324–20796912	CASP-like protein
*Csa6G445230*	20807729–20815381	Ethylene insensitive 2

### Gene Expression Pattern Analysis of the Three Candidate Genes

**Figure 5:**
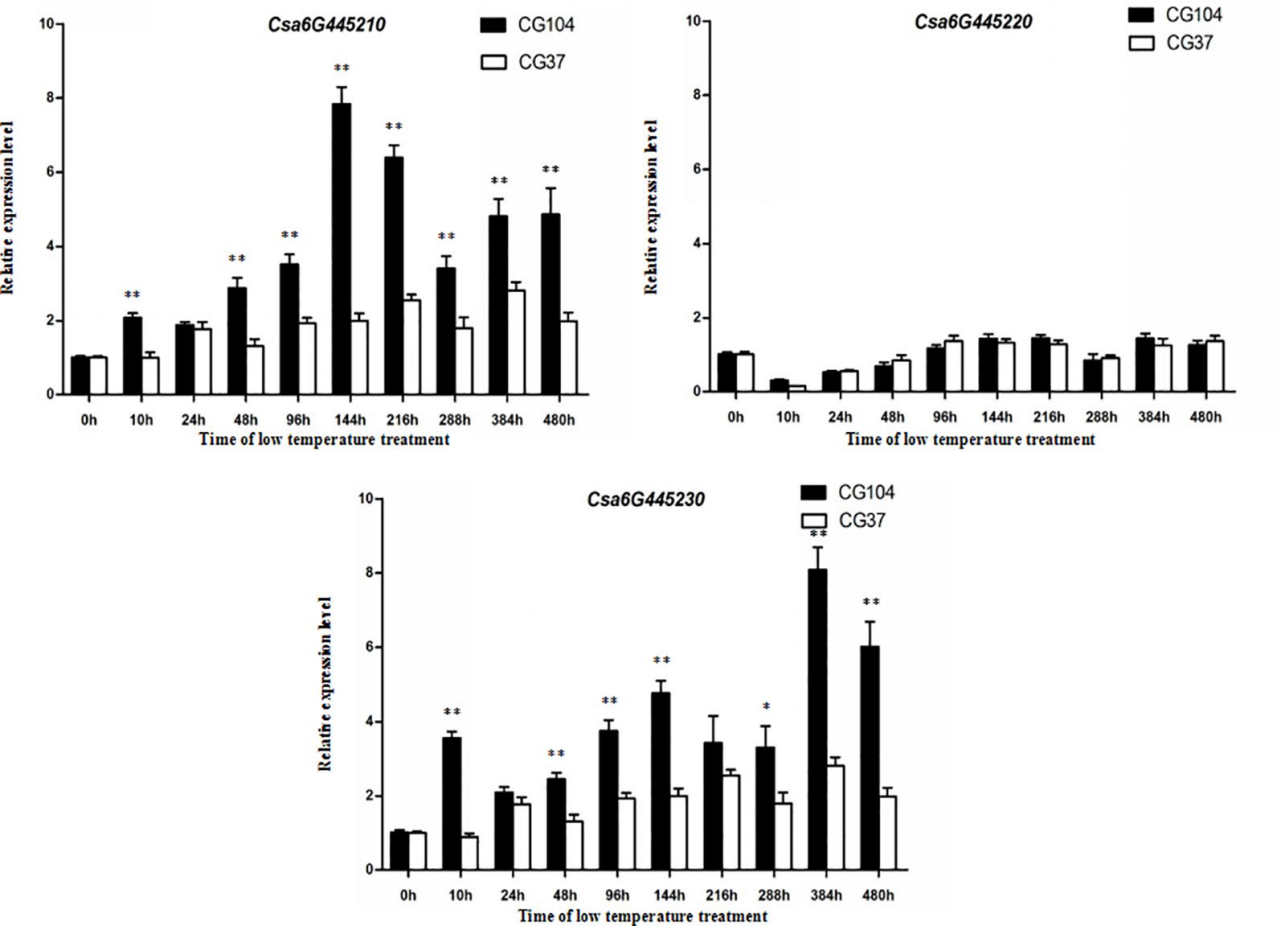
Expression analysis of three candidate genes. The fold-change indicates the relative amount of gene transcripts at different time-points compared to 0 h. The asterisks indicate that there were significant differences in transcript level between “CG104” and “CG37” (“*,” 0.01 < *P* < 0.05; “**,” 0.001 < *P* < 0.01; “***,”0 < *P* < 0.001).

To study the expression pattern of the three candidate genes under LT stress, leaf tissues of the two parental lines exposed to LT stress for 0, 10, 24, 48, 96, 144, 216, 288, 384, and 480 h were harvested, respectively, and then qRT-PCR of each gene was performed (**Figure 5**). The expression level of *Csa6G445210* in “CG104” was significantly higher than that of “CG37” at most time-points, and *Csa6G445230* showed a similar expression pattern. However, there was no significant expression difference of *Csa6G445220* between the two parental lines. In short, *Csa6G445220* did not respond to LT stress, however, both *Csa6G445210* and *Csa6G445230* were up-regulated by LT stress in “CG104.”

### Cloning and Sequence Analysis of Candidate Genes

**Figure 6:**
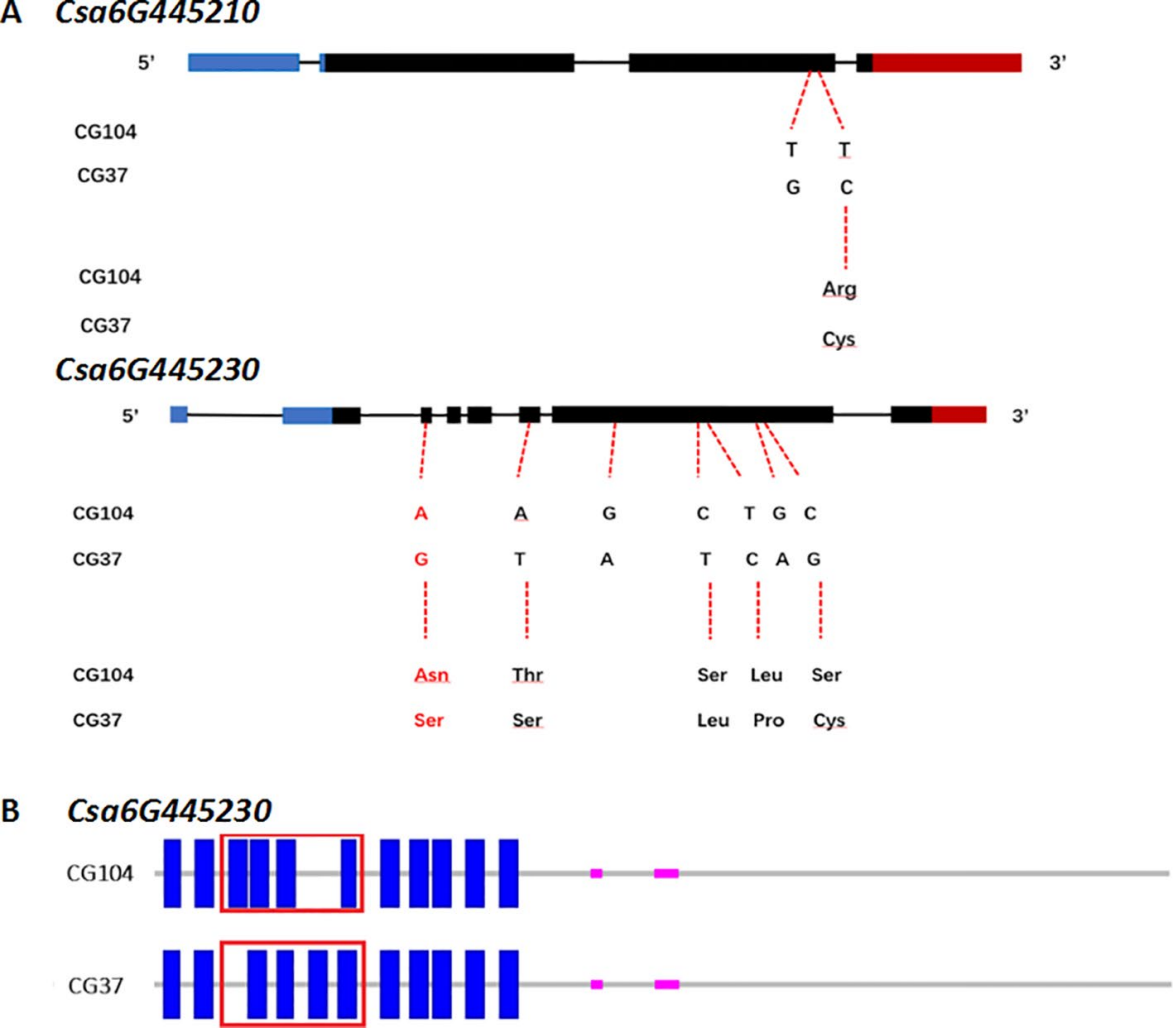
Sequence analysis of *Csa6G445210* and *Csa6G445230*. **(A)** DNA and predicted amino acid changes between parental lines. Nucleic acid indicates the SNPs on exon of *Csa6G445210* and *Csa6G445230* between “CG104” and “CG37,” and the consequential amino acid changes. The amino acid in red leads to the changes of protein secondary structure. **(B)** The secondary structure of protein encoded by *Csa6G445230* in “CG104” and “CG37.” The blue solid rectangle indicates the transmembrane helix. The red box indicates the different transmembrane structure between “CG104” and “CG37.”

To further analyze the sequence of *Csa6G445210* and *Csa6G445230*, full-length DNA and cDNA of each gene in the two parental lines were sequenced and compared (**Figure 6**, **Figures S3** and **S4**). The results showed that two base pair substitutions were detected within the coding sequence region (CDS) in *Csa6M445210*. The first substitution at position 1733 did not change the amino acid, however a polymorphism at position 1807 resulted in a non-synonymous change (Arg→Cys). Csa6M445230 has seven base pair substitutions within the CDS. Two substitutions were synonymous resulting in no amino acid change, while the rest were non-synonymous (Asp257→Ser, Thr820→Ser, Ser2468→Leu, Leu2678→Pro, Ser3260→Cys). The Asp257 resulted in changes in the transmembrane helix (**Table S8**).

The consistency between the LT tolerance phenotype, and the genotype of non-synonymous SNP sites within these two genes were examined in F_2_ population. Primers were designed based on the non-synonymous SNP site of *Csa6G445210* and the first base substitution mutation site of *Csa6G445230*. By comparing the genotype and LTII value of each F_2_ individual, it was found that both non-synonymous SNP sites within *Csa6G445210* and *Csa6G445230* were associated with the LT tolerance phenotypes (**Table S9**).

### Phylogenetic Analysis of *Csa6G445210* and *Csa6G445230*

**Figure 7:**
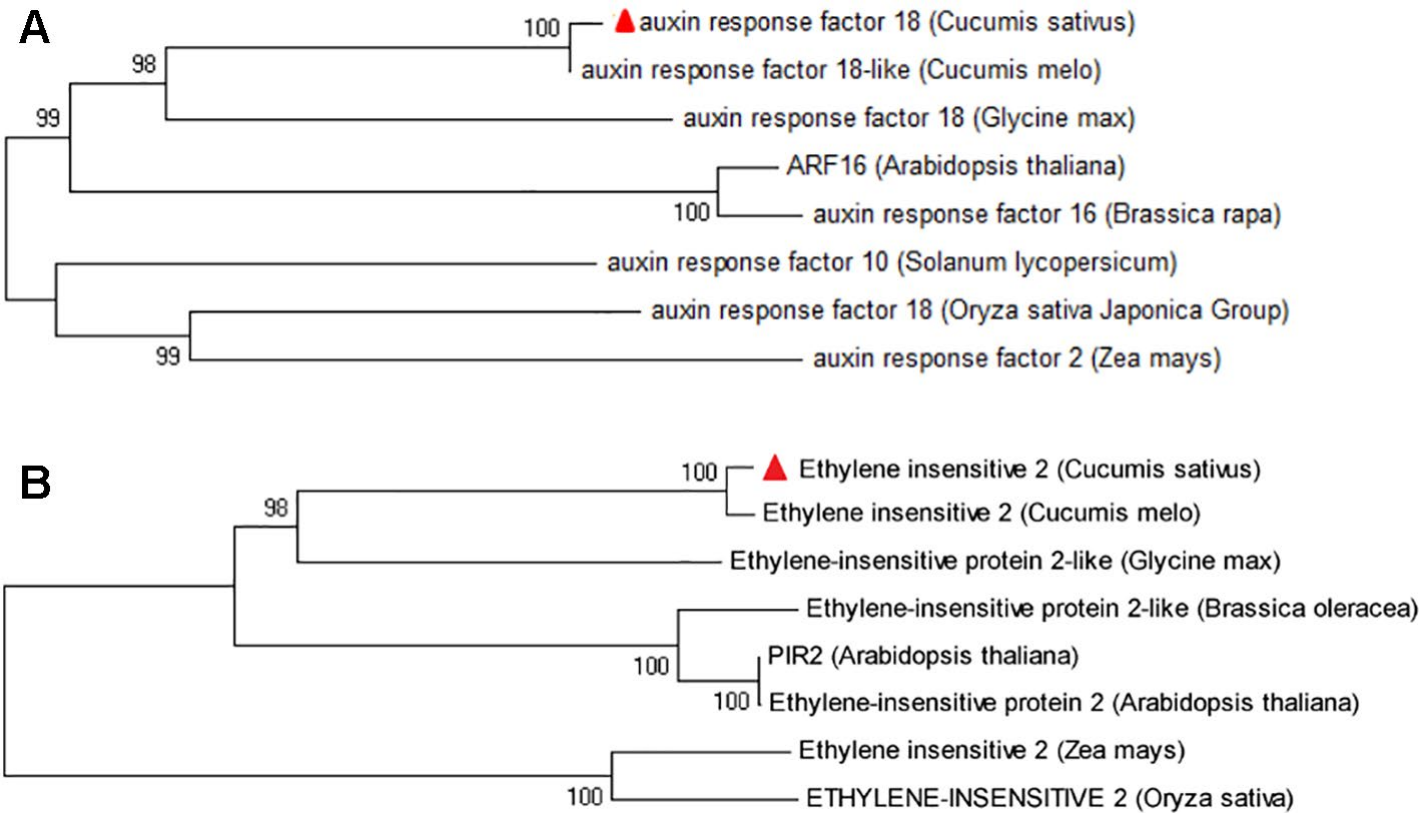
Phylogenetic tree of protein encoded by *Csa6G445210* and *Csa6G445230*. The protein encoded by cucumber *Csa6G445210* **(A)** or *Csa6G445230* **(B)** is shown as a red triangle; the branches refer to the rates of amino acid variation.

The gene annotation suggests that *Csa6M445210* encodes an auxin response factor (ARF), which is a transcription factor that controls the expression of auxin responsive genes. *Csa6M445230* encodes a transmembrane protein, ethylene-insensitive 2 (EIN2). To further predict and analyze the function of these two predicted amino acids, the sequences analyzed by BLAST against the NCBI database, and phylogenetic analyses were performed with their orthologues in seven other species (**Figure 7**). The results showed that the homologs of protein encoded by *Csa6M445210* and *Csa6G445230* are highly conserved in *A. thaliana* and other plant species, which suggests that they may share similar functions.

## Discussions

In our study, the LT stress treatments were carried out under greenhouse conditions in early spring, a natural LT environment in cucumber production. Using the yellowing degree of the first true leaf and the cotyledons as indicators, three QTLs were repeatedly identified on chromosome 5 and 6. The major QTL *qLTT6.2* was mapped into a 595-kb interval between markers SSR14859 and SSR21885 with LODs support score of 18.2 and 16.8, and explained 26.8 and 24.1% of the observed phenotypic variations. Several studies on QTL mapping of LT-tolerance in cucumber seedlings have been reported. However, the results differed due to variation in the developmental stage of the plant materials used, differences in treatment conditions including location (environmental controlled facility or field) and temperature (12 or 4°C), and different evaluation standards (degree of yellowing, area of dehydration spot, or area of dryness after recovery). Li (2014) used a CII as indicator, identified a marker SSR07248 that was closely linked to a LT-tolerant locus on Chr.6. Zhou (2015) identified six QTLs associated wtih LT tolerance on chromosome 3, and Wang (2014) identified three QTLs on chromosome 3 and one QTL on chromosome 7 using CII and a recovery index as indicators of LT-stress. However, the flanking markers were not close enough, and the contribution rate of the QTLs was low in above studies. SSR01331, the flanking maker of *qLTT6.1* identified in our study, was only 233 kb away from SSR07248 identified in the previous study (Li, 2014). The relationship between these two loci needs to be further investigated.

“*In silico* BSA” is an effective method to discover or refine markers linked to target genes or QTLs, by inspecting SNP variation among genotyped individuals at specific genomic regions. It has been used successfully in soybean to map virus resistant genes (Bello et al., 2014). With this method in cucumber, Li et al. (2016) quickly narrowed the interval around the gene controlling carpel number from a 1.9 Mb region down to 16 kb. Bo et al. (2019) also used this method to fine-map the fruit spine density related major QTL (*qfsd6.2*) to a 50 kb region, and finally identified the candidate gene *Csgl3* for the trait. In our study, the phenotypic data obtained in two replicated experiments were consistent, and the genotype sequencing depth reached 30 X, therefore this method was employed to narrow the target region. Based on the sequence data and LT-tolerance performance of CG, the genomic region of *qLTT6.2* was delimited to a 42-kb region, which only contained three candidate genes. Gene sequence alignments revealed that there were amino acid substitutions in both *Csa6G445210* and *Csa6G445230*. Furthermore, allelic diversity of the candidate gene region in F_2_ individuals showed that the two markers based on the non-synonymous SNP sites within *Csa6G445210* and *Csa6G445230* were both associated with the phenotypes of F_2_ individuals. Therefore, our study demonstrated that the *in silico* BSA approach can accurately identify SNPs linked to LT tolerance trait in cucumber.

LT is one of the major abiotic stresses that affect plant growth, development, and productivity. The mechanisms plants have evolved to adapt to LT stress have been well-studied over the past two decades (Ding et al., 2019), however, little is known in cucumber. In our study, two candidate genes *Csa6G445210* and *Csa6G445230* that might be involved in LT-stress response in cucumber seedlings were identified. *Csa6G445230* encodes EIN2 which is a key regulator in ethylene signaling pathway, and *Csa6G445210* encodes ARF which is responsive to auxin. The role of ethylene in abiotic stress resistance including cold responses was established (Yoo et al., 2009; Shi et al., 2012). In *Arabidopsis*, EIN2 is a positive regulator of the ethylene signaling pathway, indirectly inhibiting the expression of *CBF*s, which is achieved by the downstream transcription factor EIN3/EILs (Guo and Ecker, 2003; An et al., 2010; Shi et al., 2012; Li et al., 2015). CBFs could quickly activate the set of downstream cold-responsive genes *COR* (i.e., cold regulated genes), to improve plant cold tolerance (Gilmour et al., 1998; Medina et al., 1999; Miura and Furumoto, 2013; Barrero-Gil and Salinas, 2017; Shi et al., 2018). However, whether ethylene has a positive or negative effect on cold tolerance might vary among plant species; ethylene may positively regulate a low-temperature response in chilling-sensitive species (Eremina et al., 2016). In *A. thaliana*, ethylene content decreased when exposed to LT, and the loss-of-function mutant *ein2–5* was more resistant to LT stress than the wild-type plants, which indicates that ethylene negatively regulates LT stress response in *Arabidopsis* (Shi et al., 2012). The same conclusions were drawn in other LT-tolerant plants such as *Medicago sativa* (Zhao et al., 2014) and *Triticum aestivum* (Field, 1984). However, in fruit of *C. sativus* (Wang and Adams, 1982) and *S. lycopersicum* (Zhao et al., 2009), studies showed that ethylene content increased during rewarming stage after LT treatment. Whether ethylene content increased during LT treatment in leaves is yet unknown. In addition, *CsEIN2* was up-regulated in the LT-tolerant line in our study, we thus speculate that *CsEIN2* might positively regulate *CBF* in cucumber seedlings, which deserves further investigation. Although auxin is a master regulator of plant growth and development, little is known regarding its role in cold stress response. Aux/IAA binds to ARF (auxin-responsive factor) and prevents the ARF-mediated expression of downstream genes (Lavy and Estelle, 2016). A recent study showed that several DREB/CBFs genes regulate the Aux/IAA genes directly, which demonstrate that the cold responsive pathway interacts with the auxin gene regulatory network (Shani et al., 2017). However, if and how *Csa6G445210* regulates aspects of the cold tolerance signaling pathway should be determined.

## Data Availability Statement

The accession numbers of the ten core germplasm lines used in our study can be found in **Table S1**. The link of the sequence data in NCBI Short Read Archive (SRA) SRA056480 is as follows: (https://www.ncbi.nlm.nih.gov/sra/?term=SRA056480). All SNPs within the 42 kb target region can be found in **Table S3**.

## Author Contributions

WW and SW conducted the experiments and analyzed the data. SD, WW and KB analyzed the data and drafted the manuscript. ZS helped collect the data. HM helped analyze the data. XG and SZ designed the experiments.

## Funding

This research was supported by National Key Research and Development Program of China (2018YFD1000800), the Earmarked Fund for Modern Agro-industry Technology Research System (CARS-25), Science and Technology Innovation Program of the Chinese Academy of Agricultural Science (CAAS-ASTIP-IVFCAAS), Key Laboratory of Biology and Genetic Improvement of Horticultural Crops, Ministry of Agriculture, P.R. China, and Central Public-Interest Scientific Institution Basal Research Fund (No.Y2017PT52).

## Conflict of Interest

The authors declare that the research was conducted in the absence of any commercial or financial relationships that could be construed as a potential conflict of interest.
