# Supplementary material for: Quantitative Trait Loci Mapping and Candidate Gene Analysis of Low Temperature Tolerance in Cucumber Seedlings
Source: Front Plant Sci. 2019 Dec 11;10:1620. doi: 10.3389/fpls.2019.01620 (PMC6917663; doi:10.3389/fpls.2019.01620)
Supplement: Supplementary file 1 [file DataSheet_1.pdf]

Supplementary Material

1 Supplementary Tables

Supplementary Table S1. Information of ten re-sequenced varieties used in the study. R: low temperature resistant. S: low temperature sensitive.

Individual code[a]	Individual code[b]	Accession Name	Accession Number[c]	Geographical origin	Low temperature phenotype	Low temperature injury index(LTII)
CG104(P_1)	CG3087	Sekino No. 2 (Ochiai No.2)	SRX174877	Osaka, Japan	R	5.7±1.2
CG29	CG3010	Sagami Hanpaku Fushinari Kyuri	SRX174872	Japan	R	4.3±0.8
CG56	CG4358	K2148	SRX174887	Southern China	R	5.4±1.1
CG61	CG3147	185	-	Japan	R	6.3±1.0
CG90	CG1077	Qing Pi Ba Cha	SRX174857	China, Jilin	R	4.2±0.3
CG37(P_2)	CG5479	Muromskij	SRX174897	Russian Federation	S	57.8±2.9
CG10	CG7023	Rasht	SRX174917	Iran	S	63.3±1.9
CG21	CG8160	Poona Kheera	SRX174930	India, Madhya Pradesh	S	62.8±2.1
CG43	CG6562	SC 53-B (6)	SRX174908	United States	S	59.7±0.8
CG109	CG5232	Nemet Kigyo	SRX174892	Hungary	S	60.2±3.6

[a]CG # in this study; [b]CG # in Qi et al. (2013); [c]Accession number in NCBI database.

Supplementary Table S2. Environment used for evaluating low temperature tolerance of $F_{2:3}$ populations and core germplasms.

Population	Treatment	Seedling ege	Duration of low temperature	Average temperature	Site
$F_{2:3}$	March 2017	Three-week-old	11 days	15.6℃	Greenhouse
	April 2017	Three-week-old	14 days	16.6℃	Plastic greenhouse
Core germplasms	February 2016	Three-week-old	14 days	12.0℃	Greenhouse
	March 2016	Three-week-old	11 days	19.3℃	Greenhouse

Supplementary Table S3. 214 non-synonymous SNPs in the 42-kb region among 10 CG lines.

rs#	position	CG10	CG21	CG37	CG43	CG109	CG29	CG56	CG61	CG90	CG104
SNP3031244	20779616	TT	TT	TT	TT	TT	AA	AA	AA	AA	AA
SNP3031247	20779823	AA	AA	AA	AA	AA	CC	CC	CC	CC	CC
SNP3031251	20779851	CC	CC	CC	CC	CC	GG	GG	GG	GG	GG
SNP3031257	20780091	GG	GG	GG	GG	GG	TT	TT	TT	TT	TT
SNP3031258	20780095	AA	AA	AA	AA	AA	CC	CC	CC	CC	CC
SNP3031261	20780229	CC	CC	CC	CC	CC	AA	AA	AA	AA	AA
SNP3031264	20780245	AA	AA	AA	AA	AA	GG	GG	GG	GG	GG
SNP3031265	20780286	CC	CC	CC	CC	CC	GG	GG	GG	GG	GG
SNP3031272	20780688	CC	CC	CC	CC	CC	TT	TT	TT	TT	TT
SNP3031274	20780750	CC	CC	CC	CC	CC	GG	GG	GG	GG	GG
SNP3031280	20780921	AA	AA	NN	AA	AA	TT	TT	TT	TT	TT
SNP3031281	20780926	AA	AA	AA	AA	AA	CC	CC	CC	CC	CC

SNP3031282	20780947	CC	NN	CC	CC	CC	AA	AA	AA	AA	AA
SNP3031283	20780962	AA	NN	AA	AA	AA	CC	CC	CC	CC	CC
SNP3031284	20780974	CC	CC	CC	CC	CC	TT	NN	TT	TT	TT
SNP3031285	20781002	AA	AA	AA	AA	AA	CC	CC	CC	CC	CC
SNP3031287	20781044	TT	TT	TT	TT	TT	CC	CC	CC	CC	CC
SNP3031290	20781161	AA	AA	AA	AA	AA	CC	CC	CC	CC	CC
SNP3031291	20781182	GG	GG	GG	NN	GG	AA	AA	AA	AA	AA
SNP3031327	20782412	TT	TT	TT	NN	TT	GG	GG	GG	GG	GG
SNP3031355	20784113	TT	TT	TT	TT	TT	GG	GG	GG	GG	GG
SNP3031356	20784184	CC	CC	CC	CC	CC	AA	AA	AA	AA	AA
SNP3031364	20784298	TT	TT	TT	TT	TT	GG	GG	GG	GG	GG
SNP3031366	20784321	TT	TT	TT	TT	TT	GG	GG	GG	GG	GG
SNP3031368	20784367	TT	TT	TT	TT	TT	GG	GG	GG	GG	GG
SNP3031369	20784380	AA	AA	AA	AA	AA	CC	CC	CC	CC	CC
SNP3031372	20784406	AA	AA	NN	AA	AA	TT	TT	TT	TT	TT
SNP3031375	20784475	AA	AA	GA	AA	AA	CC	CC	CC	CC	CC
SNP3031376	20784500	GG	GG	GC	GG	GG	CC	CC	CC	CC	CC
SNP3031377	20784510	GG	GG	GG	GG	GG	TT	TT	TT	TT	TT
SNP3031382	20784694	GC	CC	CC	CC	CC	GG	GG	GG	GG	GG
SNP3031383	20784700	GC	CC	CC	CC	CC	GG	GG	GG	GG	GG
SNP3031384	20784793	AA	AA	AA	AA	AA	CC	CC	CC	CC	CC
SNP3031385	20784805	AA	AA	AA	AA	AA	CC	CC	CC	CC	CC
SNP3031386	20784866	TT	TT	TC	TT	TT	GG	GG	GG	GG	GG
SNP3031393	20785203	AA	AA	AA	AA	AA	CC	CC	CC	CC	CC
SNP3031394	20785226	TT	TT	TT	TT	TT	GG	GG	GG	GG	GG
SNP3031396	20785258	TT	TT	NN	TT	TT	AA	AA	AA	AA	AA
SNP3031403	20785465	CC	CC	CC	CC	CC	AA	AA	AA	AA	AA
SNP3031406	20785675	TT	TT	TT	TT	TT	GG	GG	GG	GG	GG
SNP3031411	20786082	TT	TT	TT	TT	TT	GG	GG	GG	GG	GG
SNP3031412	20786264	GG	GG	GG	GG	GG	CC	CC	CC	CC	CC
SNP3031422	20787866	TT	TT	TT	TT	TT	AA	AA	AA	AA	AA
SNP3031423	20787899	GG	GG	GG	GG	GG	TT	TT	TT	TT	TT
SNP3031424	20788611	CC	CC	GT	CC	CC	TT	TT	TT	TT	TT
SNP3031425	20788645	GG	GG	GG	GG	GG	TT	TT	TT	TT	TT
SNP3031427	20788941	CC	CC	CC	CC	CC	TT	TT	TT	TT	TT
SNP3031441	20789893	CC	CC	CC	CC	CC	AA	AA	AA	AA	AA
SNP3031453	20790302	AA	AA	AA	NN	AA	CC	CC	CC	CC	CC
SNP3031454	20790362	TT	TT	TT	NN	TT	AA	AA	AA	AA	AA
SNP3031455	20790377	AA	AA	AA	NN	AA	CC	CC	CC	CC	CC
SNP3031458	20790634	TT	TT	TT	TT	TT	AA	NN	AA	AA	AA
SNP3031467	20790930	CC	CC	CC	CC	CC	AA	AA	AA	AA	AA
SNP3031468	20790965	GG	NN	GG	GG	GG	TT	TT	TT	TT	TT
SNP3031469	20790967	TT	NN	TT	TT	TT	GG	GG	TC	GG	GG
SNP3031471	20791187	GG	GG	GG	GG	GG	AA	AA	AA	AA	AA
SNP3031472	20791323	CC	CC	CC	CC	CC	AA	AA	AA	AA	AA
SNP3031473	20791339	TT	TT	TT	TT	TT	AA	AA	AA	AA	AA
SNP3031491	20791602	CC	CC	NN	CC	CC	AA	AA	AA	AA	AA
SNP3031492	20791626	TT	TT	NN	TT	TT	AA	AA	NN	AA	AA

SNP3031493	20791650	CC	CC	CC	CC	CC	AA	NN	AA	AA	AA
SNP3031494	20791665	CC	CC	CC	CC	CC	AA	AA	AA	AA	AA
SNP3031495	20791675	GG	GG	GG	GG	GG	AA	AA	AA	AA	AA
SNP3031496	20791688	GG	GG	GG	GG	GG	TT	TT	TT	TT	TT
SNP3031499	20791804	GG	GG	GG	GG	GG	TT	TT	TT	TT	TT
SNP3031500	20791943	TT	TT	TT	TT	TT	GG	GG	GG	GG	GG
SNP3031503	20792076	AA	AA	NN	AA	AA	CC	CC	CC	CC	CC
SNP3031504	20792099	AA	AA	AA	AA	AA	GG	GG	GG	GG	GG
SNP3031512	20792491	GG	GG	GG	GG	GG	AA	AA	NN	AA	AA
SNP3031528	20793291	TT	TT	TT	TT	TT	GG	GG	GG	GG	GG
SNP3031529	20793303	TT	TT	TT	TT	TT	AA	AA	AA	AA	AA
SNP3031531	20793355	TT	TT	TT	TT	TT	GG	GG	GG	GG	GG
SNP3031534	20793407	CC	CC	CC	CC	CC	GG	GG	GG	GG	GG
SNP3031535	20793451	TT	TT	TT	TT	TT	GG	GG	GG	GG	GG
SNP3031536	20793468	CC	CC	CC	CC	CC	AA	AA	AA	AA	AA
SNP3031538	20793507	GG	GG	GG	GG	GG	CC	CC	CC	CC	CC
SNP3031540	20793568	CC	CC	CC	CC	CC	TT	TT	TT	TT	TT
SNP3031550	20793963	TT	TT	TT	TT	TT	TT	TT	NN	TT	TT
SNP3031556	20794115	CC	CC	CC	CC	CC	TT	TT	TT	TT	TT
SNP3031560	20794168	CC	CC	CC	CC	CC	AA	AA	AA	AA	AA
SNP3031564	20794262	TT	TT	TT	TT	TT	AA	AA	AA	AA	AA
SNP3031571	20794941	TT	TT	TT	TT	TT	GG	GG	GG	GG	GG
SNP3031572	20795196	TT	TT	TT	TT	TT	GG	GG	GG	GG	GG
SNP3031575	20795310	GG	GG	GG	GG	GG	TT	TT	TT	TT	TT
SNP3031576	20795315	CC	CC	CC	CC	CC	TT	TT	TT	TT	TT
SNP3031580	20795444	AA	AA	AA	AA	AA	CC	CC	CC	CC	CC
SNP3031584	20795680	CC	CC	CC	CC	CC	TT	TT	TT	TT	TT
SNP3031585	20795739	AA	AA	AA	AA	AA	CC	CC	CC	CC	CC
SNP3031588	20795768	CC	CC	NN	CC	CC	AA	AA	AA	AA	AA
SNP3031589	20795883	GG	GG	GG	GG	GG	TT	TT	TT	TT	TT
SNP3031592	20796005	AA	AA	AA	AA	AA	CC	CC	CC	CC	CC
SNP3031593	20796066	TT	TT	TT	TT	TT	GG	GG	GG	GG	GG
SNP3031598	20796164	CC	CC	CC	CC	CC	AA	AA	AA	AA	AA
SNP3031599	20796183	GG	GG	GG	GG	GG	TT	TT	TT	TT	TT
SNP3031605	20796885	TT	TT	TT	TT	TT	GG	GG	GG	GG	GG
SNP3031606	20796914	TT	TT	TT	TT	TT	GG	GG	GG	GG	GG
SNP3031607	20796931	GG	GG	GG	GG	GG	TT	TT	TT	TT	TT
SNP3031612	20797272	CC	CC	CC	CC	CC	TT	TT	TT	TT	TT
SNP3031614	20797331	CC	CC	CC	CC	CC	TT	TT	TT	TT	TT
SNP3031615	20797363	TT	TT	TT	TT	TT	GG	GG	GG	GG	GG
SNP3031616	20797378	GG	GG	GG	GG	GG	TT	TT	TT	TT	TT
SNP3031621	20797492	AA	AA	AA	AA	AA	CC	CC	CC	CC	CC
SNP3031633	20798249	GG	GG	GG	GG	GG	CC	CC	CC	CC	CC
SNP3031642	20798572	AA	AA	AA	AA	AA	CC	CC	CC	CC	CC
SNP3031644	20798602	AA	AA	AA	AA	AA	CC	CC	CC	CC	CC
SNP3031645	20798656	TT	TT	TT	TT	TT	GG	GG	GG	GG	GG
SNP3031648	20798687	TT	TT	TT	TT	TT	CC	CC	CC	CC	CC
SNP3031650	20798707	TT	TT	TT	TT	TT	GG	GG	GG	GG	GG

SNP3031653	20798807	TT	TT	TT	TT	TT	GG	GG	GG	GG	GG
SNP3031654	20798822	AA	AA	AA	AA	NN	TT	TT	TT	TT	TT
SNP3031655	20798858	CC	CC	NN	CC	CC	GG	GG	GG	GG	GG
SNP3031658	20799027	AA	AA	AA	AA	AA	CC	CC	CC	CC	CC
SNP3031661	20799180	AA	AA	NN	AA	AA	GG	GG	GG	GG	GG
SNP3031669	20799304	GG	GG	GG	GG	GG	TT	TT	TT	TT	TT
SNP3031671	20799384	GG	GG	NN	GG	GG	TT	TT	TT	TT	TT
SNP3031676	20799480	TT	TT	TT	TT	TT	GG	GG	GG	GG	GG
SNP3031679	20799523	GG	GG	GG	GG	GG	TT	TT	TT	TT	TT
SNP3031680	20799526	GG	GG	GG	GG	GG	TT	TT	TT	TT	TT
SNP3031683	20799665	GG	GG	GG	GG	GG	TT	TT	TT	TT	TT
SNP3031688	20799905	CC	CC	CC	CC	CC	GG	GG	GG	GG	GG
SNP3031696	20800166	AA	NN	AA	AA	AA	CC	CC	CC	CC	CC
SNP3031697	20800218	AA	AA	AA	AA	AA	GG	GG	GG	GG	GG
SNP3031698	20800382	CC	CC	CC	CC	CC	AA	AA	AA	AA	AA
SNP3031701	20800729	TT	TT	NN	TT	TT	CC	CC	CC	CC	CC
SNP3031702	20800816	CC	CC	CC	CC	CC	AA	AA	AA	AA	AA
SNP3031703	20800818	CC	CC	CC	CC	CC	AA	AA	AA	AA	AA
SNP3031715	20801363	CC	CC	CC	CC	CC	AA	AA	AA	AA	AA
SNP3031716	20801446	TT	TT	TT	TT	TT	AA	AA	AA	AA	AA
SNP3031722	20801801	TT	TT	TT	TT	TT	CC	CC	CC	CC	CC
SNP3031726	20802633	TT	TT	TT	TT	TT	AA	AA	NN	AA	AA
SNP3031732	20802778	TT	TT	NN	TT	TT	GG	GG	NN	GG	GG
SNP3031747	20803301	NN	AA	AA	AA	AA	CC	CC	CC	CC	CC
SNP3031749	20803317	GG	GG	NN	GG	GG	TT	TT	TT	TT	TT
SNP3031750	20803342	TT	TT	NN	TT	TT	AA	AA	AA	AA	AA
SNP3031751	20803350	GG	GG	NN	GG	GG	TT	TT	TT	TT	TT
SNP3031753	20803390	CC	CC	NN	CC	CC	GG	GG	GG	GG	GG
SNP3031754	20803400	TT	TT	NN	TT	NN	AA	AA	AA	AA	AA
SNP3031761	20803738	CC	CC	CC	CC	CC	TT	TT	NN	TT	TT
SNP3031762	20803742	TT	TT	TT	TT	TT	GG	GG	NN	GG	GG
SNP3031765	20803920	AA	AA	AA	AA	AA	TT	TT	TT	TT	TT
SNP3031766	20804002	TT	TT	TT	TT	TT	GG	GG	GG	GG	GG
SNP3031767	20804007	GG	GG	GG	GG	GG	TT	TT	TT	TT	TT
SNP3031775	20804618	AA	AA	AA	AA	AA	CC	CC	CC	CC	CC
SNP3031776	20804704	GG	GG	GG	GG	GG	TT	TT	TT	TT	TT
SNP3031779	20805032	TT	TT	TT	TT	TT	AA	AA	AA	AA	AA
SNP3031780	20805053	TT	TT	TT	TT	TT	AA	AA	AA	AA	AA
SNP3031781	20805066	NN	CC	CC	CC	CC	TT	TT	TT	TT	TT
SNP3031783	20805084	AA	AA	AA	AA	AA	CC	CC	CC	CC	CC
SNP3031785	20805138	CC	CC	CC	CC	CC	TT	TT	TT	TT	TT
SNP3031791	20805585	GG	GG	GG	GG	GG	TT	TT	TT	TT	TT
SNP3031797	20805881	GG	GG	GG	GG	GG	CC	CC	CC	CC	CC
SNP3031798	20805917	CC	CC	CC	CC	CC	AA	AA	AA	AA	AA
SNP3031799	20805922	CC	CC	CC	CC	CC	AA	AA	AA	AA	AA
SNP3031802	20805991	AA	AA	AA	AA	AA	CC	CC	CC	CC	CC
SNP3031808	20806215	CC	CC	CC	CC	CC	GG	GG	GG	GG	GG
SNP3031809	20806332	CC	CC	CC	CC	CC	TT	TT	TT	TT	TT

SNP3031813	20806430	GG	GG	GG	GG	GG	TT	TT	TT	TT	TT
SNP3031818	20807378	CC	CC	CC	CC	CC	AA	NN	AA	AA	AA
SNP3031819	20807440	AA	AA	AA	AA	AA	TT	TT	TT	TT	TT
SNP3031820	20807664	GG	GG	GG	GG	GG	AA	AA	NN	AA	AA
SNP3031823	20807859	AA	AA	AA	AA	AA	CC	CC	CC	CC	CC
SNP3031824	20807902	GG	GG	GG	GG	GG	TT	TT	TT	TT	TT
SNP3031832	20808197	AA	AA	AA	AA	AA	CC	CC	CC	CC	CC
SNP3031833	20808218	AA	AA	AA	AA	AA	TT	TT	TT	TT	TT
SNP3031837	20808492	AA	AA	AA	AA	AA	CC	CC	CC	CC	CC
SNP3031839	20808631	CC	CC	CC	CC	CC	AA	AA	AA	AA	AA
SNP3031840	20808730	GG	GG	GG	GG	GG	TT	TT	TT	TT	TT
SNP3031843	20808767	CC	CC	CC	CC	CC	AA	AA	AA	AA	AA
SNP3031844	20808769	GG	GG	GG	GG	GG	CC	CC	CC	CC	CC
SNP3031846	20809016	GG	GG	GG	GG	GG	TT	TT	TT	TT	TT
SNP3031851	20809513	AA	AA	AA	AA	AA	TT	TT	TT	TT	TT
SNP3031853	20809530	AA	AA	AA	AA	AA	GG	GG	GG	GG	GG
SNP3031856	20809681	TT	TT	TT	TT	TT	GG	GG	GG	GG	GG
SNP3031857	20809688	CC	CC	CC	CC	CC	AA	AA	AA	AA	AA
SNP3031858	20809752	CC	CC	CC	CC	CC	AA	AA	AA	AA	AA
SNP3031861	20810087	CC	CC	CC	CC	CC	AA	NN	AA	AA	AA
SNP3031864	20810465	GG	GG	GG	GG	GG	TT	TT	TT	TT	TT
SNP3031867	20811160	TT	TT	TT	TT	TT	AA	AA	AA	AA	AA
SNP3031874	20812261	AA	AA	AA	AA	AA	CC	CC	CC	CC	CC
SNP3031879	20812926	TT	TT	TT	TT	TT	GG	GG	GG	GG	GG
SNP3031881	20813136	GG	GG	GG	GG	GG	TT	TT	TT	TT	TT
SNP3031883	20813632	AA	AA	AA	AA	AA	CC	CC	CC	CC	CC
SNP3031884	20813718	CC	CC	CC	CC	CC	GG	GG	GG	GG	GG
SNP3031885	20814033	CC	CC	CC	CC	CC	TT	TT	TT	TT	TT
SNP3031888	20814218	CC	CC	CC	CC	CC	AA	AA	AA	AA	AA
SNP3031890	20814314	GG	GG	GG	GG	GG	AA	AA	AA	AA	AA
SNP3031891	20814904	CC	CC	CC	CC	CC	AA	AA	AA	AA	AA
SNP3031892	20815099	CC	CC	CC	CC	CC	AA	AA	AA	AA	AA
SNP3031896	20815615	AA	AA	AA	AA	AA	TT	TT	TT	TT	TT
SNP3031906	20816191	CC	NN	CC	CC	CC	GG	GG	GG	GG	GG
SNP3031907	20816195	CC	NN	CC	CC	CC	GG	GG	GG	GG	GG
SNP3031910	20816305	GG	GG	GG	GG	GG	TT	TT	TT	TT	TT
SNP3031911	20816348	AA	AA	AA	AA	AA	CC	CC	CC	CC	CC
SNP3031917	20816402	TT	TT	TT	TT	TT	GG	GG	GG	GG	GG
SNP3031918	20816412	TT	TT	TT	TT	NN	GG	GG	GG	GG	GG
SNP3031938	20816802	CC	CC	NN	CC	CC	TT	NN	TT	TT	TT
SNP3031939	20816906	TT	TT	TT	TT	TT	NN	AA	AA	AA	AA
SNP3032015	20820005	GG	GG	NN	GG	GG	AA	AA	NN	AA	AA
SNP3032021	20820284	AA	AA	AA	AA	AA	CC	CC	CC	CC	CC
SNP3032022	20820290	GG	GG	GG	GG	GG	TT	TT	TT	TT	TT
SNP3032023	20820350	TT	TT	TT	TT	TT	GG	GG	NN	GG	GG
SNP3032024	20820381	GG	GG	NN	GG	GG	TT	TT	TT	TT	TT
SNP3032025	20820397	AA	AA	NN	AA	AA	CC	CC	CC	CC	CC
SNP3032031	20820481	GG	GG	GG	GG	GG	TT	TT	TT	TT	TT

SNP3032032	20820534	CC	CC	NN	CC	CC	AA	AA	AA	AA	AA
SNP3032033	20820621	GG	GG	GG	GG	GG	TT	TT	TT	TT	TT
SNP3032035	20820719	AA	AA	AA	AA	AA	TT	TT	TT	TT	TT
SNP3032040	20820994	TT	TT	TT	TT	TT	CC	CC	CC	CC	CC
SNP3032043	20821125	TT	TT	TT	TT	TT	AA	AA	AA	AA	AA
SNP3032044	20821153	CC	CC	CC	CC	CC	GG	GG	GG	GG	GG
SNP3032047	20821287	GG	GG	GG	GG	GG	AA	AA	AA	AA	AA
SNP3032052	20821448	CC	CC	NN	CC	CC	GG	GG	GG	GG	GG
SNP3032054	20821614	CC	CC	NN	CC	CC	AA	AA	AA	AA	AA
SNP3032055	20821620	AA	AA	NN	AA	AA	TT	TT	TT	TT	TT

Supplementary Table S4. The primers used for gene cloning and gene expression analysis.

	Primer name	Forward primer	Reverse primer
Gene cloning for sequencing	*Csa6G445210-1*	TCTCTTCGCTTCTCTTCTG	TTAATTCCTCTGCCAACCA
	Csa6G445210-2	TTGCTGTTGTTAGATGGTC	GGCGTCCCTCTATAAATGT
	Csa6G445210-3	AGTCTGATGCTAACAATGGT	GTGGTAAGGTCAACTACTACA
	Csa6G445210-4	GCTTCTTCAGGTAATCACTGT	AGGAGTCTTCACGCCATC
	Csa6G445210-5	CGTATCTTGCTTGCTAACC	GAAGGACCTGATATTGACCA
	Csa6G445210-6	CATCAAGACAGCAAGAAGAT	TCCAAATACACCCATAGACT
	Csa6G445220-1	GCGAATCTTCTTCCTTACAC	TTACAAGACCTCAGCGATAG
	Csa6G445220-2	CGTTACAGAGTCAATGAACT	CAACAGCAGCAACAAGATA
	Csa6G445220-3	GCTGTTGGCTAATGTTACTC	ACACTTGTTAAGGTCATTGC
	Csa6G445220-4	AATAAGGAGTCGTGTTGTC	AAAGAAATGTCAGTGGAGTG
	Csa6G445230-1	AGCAGTCTATCACTCTCATC	CCTTACCTTACCGCCTATT
	Csa6G445230-2	TTTGGGTTCTGGGTTCTG	TGTGGTTATCTTCCTTAGCA
	Csa6G445230-3	TCCTTCTCTGATGCTAATGG	GCTGCTTGATAGACACAAC
	Csa6G445230-4	TGGTCACTGGAAGAGGTC	TGGCTGGAAGTCTGGAAT
	Csa6G445230-5	CAACGAGGAGTATGATAAGTG	CAACCGCCTAGAATGACA
	Csa6G445230-6	AATCTCATGCCGACAAGG	TGACCACCAAGACTCCAT
	Csa6G445230-7	TGGAGTCTTGGTGGTCAA	TATAGGCTCGGCACTGTAT
	Csa6G445230-8	GGATATGGCAGAGGTTAGAC	CAATAGGTGATGAGTGAGGAA
	Csa6G445230-9	GAGAAGGATGATTATGAGGTTG	CTGGATACTGTGATGTTGGA
	Csa6G445230-10	TTCCAACATCACAGTATCCA	ACAGCCTTCTCCACAATG
	Csa6G445230-11	GCTTCTCCAGTCCTTCAG	TTGCCATTAGAGTTCACAAG
	Csa6G445230-12	GTGCTTACTGGCTCCATT	GTCCATTGAGTCACATCCTA
	Csa6G445230-13	CCACTCACGAAGTATCATCT	ACGATCATATCATGGAGCAA
qRT-PCR	*Actin1*	CACCAAGCCCAAGAAGATC	TAAACCTAATCACCACCAGC
	Csa6G445210	TGTCTTGTACCGTGATGTT	GCTCCTACATTGTTACTTCC
	Csa6G445220	TATCTTGTTGCTGCTGTTG	GTAAGGGTGGACGTAACA
	Csa6G445230	AACATGATCTTGGGCATTTC	CCATCTTCCAGAAGGTCAG

Supplementary Table S5. Specific SNP markers used for amplifying mutation sites from F_2 individuals.

Gene name	Primer name	Primer sequence	Restriction endonuclease	Restriction site	Mutation site	Phenotype
Csa445210	SNP_10-F	AAACCTCTCCGATGGCTCTG	MfeI	5'...C▼AATTG...3' 3'...GTTAA▲C...5'	...CAAT**T**GCC...	R
	SNP_10-R	TGTTCGTCACCGACATGTTT			...CAAT**C**GCC...	S
Csa445230	SNP_30-F	GGTTTGTTCCATGGGTATAGGT	BbvI	5'...GCAGC ▼ $(N)_8$...3'	...GCA**A**CG...	R
	SNP_30-R	ACACATGTGAAGAGGTCCCA		3'...CGTCG▲ $(N)_{12}$...5'	...GCA**G**CG...	S

Supplementary Table S6. Low temperature injury index of F_1 and F_1' showed that the LT

tolerance is controlled by nuclear genes.

Parental Lines		F_1	F_1’
CG104	CG37	CG104 x CG37	CG37 x CG104
（Mean±S.E）		（Mean±S.E）	（Mean±S.E）
4.1±1.7	65.3±6	27±7.8	30.0±3.4

Three replicates were conducted in the experiment, and eight plants were phenotyped for each replicate.

Supplementary Table S7. Distribution of markers on the linkage map.

Chromosome	Number of markers	Length (cM)	Average distance (cM/marker)
Chr1	27	119.1	4.4
Chr2	47	160.4	3.4
Chr3	29	160.9	5.5
Chr4	18	144.7	8.0
Chr5	35	152.8	4.4
Chr6	44	163.3	3.8
Chr7	25	89.4	3.6
Total	190	990.8	5.2

Supplementary Table S8. Transmembrane helix position of protein encoded by *Csa6G445230.*

Name	CG104		CG37	
	Start	End	Start	End
transmembrane region	13	32	13	32
transmembrane region	52	74	52	74
transmembrane region	95	117	120	142
transmembrane region	122	144	157	176
transmembrane region	156	178	197	219
transmembrane region	238	255	234	256
transmembrane region	288	310	288	310
transmembrane region	325	347	325	347
transmembrane region	354	376	354	376
transmembrane region	396	418	396	418
transmembrane region	439	461	439	461
low complexity	554	569	554	569
low complexity	635	666	635	666

Supplementary Table S9. Correlation between phenotype and the non-synonymous SNP site of *Csa6G445210* (SNP_10) and *Csa6G445230* (SNP_30) in 158 F_2 individuals. The individuals that produced the same digestion products as CG104, CG37, and F_1 were defined as type a, b, and ab, respectively.

Code	Mar-17	April 2017	SNP_10	SNP_30	Code	Mar-17	April 2017	SNP_10	SNP_30
2	47.7	33.7	b	b	108	22	18.9	a	a
3	40.5	37.5	ab	ab	109	27	20.1	a	a
4	63	-	ab	ab	110	34.8	34.5	ab	ab
5	15.7	27	ab	ab	113	31.5	33.9	ab	ab
6	14.6	14.3	a	a	114	34.9	30.7	b	b
7	27	23.5	ab	ab	115	34.7	31.2	ab	ab
8	47	37.6	-	b	116	21	17.6	a	a
9	39.2	37.2	ab	ab	117	35.3	20.7	a	a

10	45	38.7	ab	ab	118	37.8	33.5	ab	ab
12	44.2	29.3	ab	ab	119	61.1	56.6	b	b
13	39.7	36.8	b	b	120	17.7	17	a	a
15	20.3	14.5	ab	ab	121	34.3	30.9	ab	ab
16	21.2	15.3	a	a	122	44.3	42.3	ab	ab
18	18.3	8.6	a	a	123	27	27.8	ab	ab
22	12.5	6.4	a	a	125	36	24.6	ab	ab
23	32.3	30	ab	ab	126	17.6	18.9	a	a
24	30.9	28.5	ab	ab	127	32	28.9	b	b
26	27	14.3	a	a	129	31	29	ab	ab
27	31	28.8	-	ab	133	35.7	35.7	b	b
28	33.9	32	ab	ab	136	38.8	37	ab	ab
29	24.1	16.7	ab	ab	139	26.3	28.5	ab	ab
30	50.3	37	b	b	140	24.8	24.8	ab	ab
31	42.3	29.7	ab	ab	145	38	34	b	b
33	61.7	46.2	b	b	146	30.3	30.7	ab	ab
34	41.9	32.7	ab	ab	147	29.8	24.2	a	a
35	25.8	19.6	ab	ab	148	38.2	29.7	ab	ab
36	25.8	21	a	a	149	45	42.9	b	b
37	14.8	5.7	ab	ab	152	49.7	45.8	b	b
39	49.3	36.8	ab	ab	153	13	15.7	a	a
40	48.2	33.9	b	b	156	33.8	27.6	ab	ab
42	13.8	11.2	a	a	157	37.6	33.2	ab	-
48	52.3	36	b	b	158	28.3	23.3	ab	ab
49	35.6	32.6	b	b	159	37.4	31.5	ab	ab
50	36	32.2	ab	ab	162	28.2	20.8	a	a
51	16.7	16.1	a	a	163	38.2	33.5	ab	ab
52	29.2	32.9	ab	ab	164	34.2	19.6	ab	ab
53	25.1	28.6	ab	ab	165	31	23.5	ab	ab
54	45	34.3	ab	ab	166	45.7	46.3	b	b
55	51	47	b	b	169	30.6	31.9	ab	ab
56	22.7	15.6	a	a	172	17.6	35.2	a	a
58	35.5	37	b	b	174	15.1	13.3	a	a
60	21.2	18.7	a	a	175	34.8	34.6	ab	ab
61	19.1	12.7	a	a	176	41.3	38.6	ab	ab
62	18.3	16.1	a	a	178	28.7	26	ab	ab
63	45	36.8	ab	ab	179	42.4	34.7	ab	ab
65	42	39	b	b	180	39	34.1	ab	ab
66	46.4	33	b	b	181	29.3	16.9	ab	ab
67	39.8	34.9	ab	ab	182	3.8	9.4	-	a
69	49.3	37.3	ab	ab	183	45.6	43.8	ab	ab
70	49.9	34.9	ab	ab	184	35.7	35.3	ab	ab
71	11.7	27	a	a	185	31.3	27.4	ab	ab
72	38.8	26.3	ab	-	186	33.6	18	ab	ab
74	18.4	14.1	a	a	187	36.4	33.6	ab	ab
77	46.2	42	b	b	188	39.2	37.9	ab	ab
80	39.2	28.9	ab	ab	189	16.2	20.3	a	a
81	28.7	30.5	ab	ab	190	19.5	12.5	a	a

82	45	34.3	ab	ab	191	33	27.8	ab	ab
83	29.5	30.6	ab	ab	192	51.2	52.3	b	b
84	23.3	23.7	a	a	193	43	24.8	ab	ab
85	24.6	24.8	ab	ab	194	13.7	16.1	a	a
86	20.3	15	a	a	195	25.8	19.2	ab	ab
87	29.8	22.8	ab	ab	197	41.1	38.1	b	b
89	33.7	28	ab	ab	198	27	25.6	ab	ab
90	53	41.9	b	b	199	27	26.2	a	a
91	31.7	29.4	ab	ab	200	31.7	37.9	ab	ab
92	37.8	34.6	ab	ab	202	38.2	37.9	b	b
93	29.6	26.3	a	a	203	35.4	35.4	b	b
94	39.2	29.3	ab	ab	205	43.6	34.8	ab	ab
95	36	33.5	ab	ab	206	25.2	19.3	ab	ab
96	25.7	21.2	a	-	209	30.2	10.5	a	a
98	14.4	21.1	a	a	210	37.2	35.7	ab	ab
99	23.7	19.7	a	a	211	30.7	23.8	a	a
101	37.4	39.8	ab	ab	212	32.3	29.9	ab	ab
102	26	26	a	a	213	27.4	25.6	ab	ab
103	18	20.5	a	a	215	31.2	30.6	ab	ab
104	22.2	23.9	a	a	217	14	15.1	a	a
105	22.3	16.9	a	a	218	46.3	33	ab	ab
106	45	45.6	ab	ab	219	16	12.3	a	a
107	30.9	27	a	a	222	23.8	27	ab	ab

2 Supplementary Figures

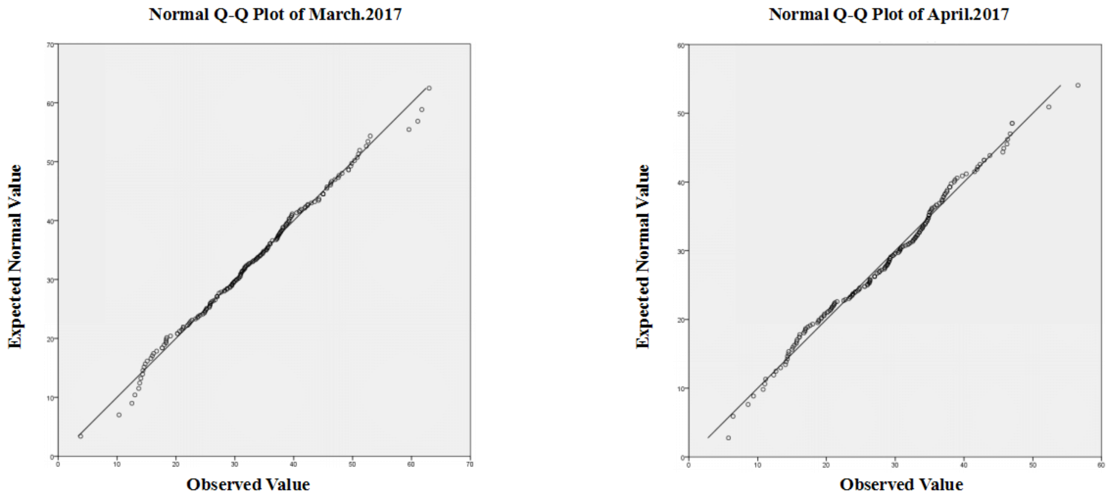

Supplementary Figure S1. The Normal Q-Q plot of phenotypic data generated in March 2017 and April 2017.

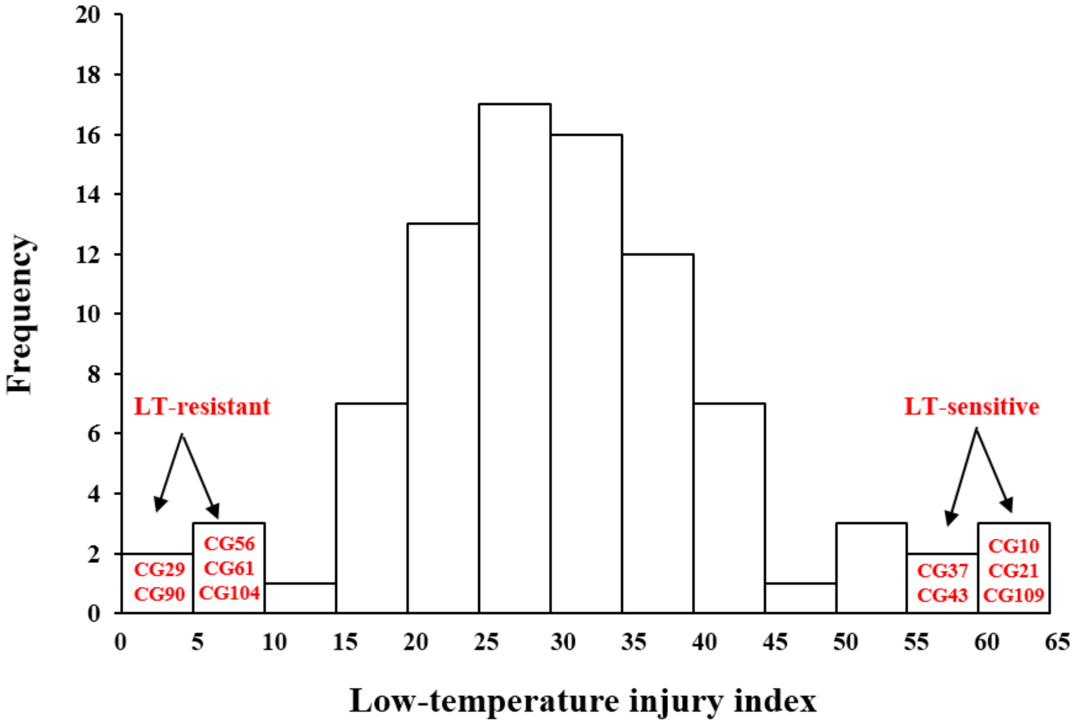

Supplementary Figure S2. The frequency distribution of the low temperature injury index of 87 core germplasm. The ten CG lines in red were selected to generate LT-resistant and LT-sensitive bulks.

Csa6G445210

```
CG104   1      CTTCTTCCCTTCTTCTCCTCTCTAGGATTCTCTTTTCCCTTCCTTCCATCAATTCCCACT   60
               ||||||||||||||||||||||||||||||||||||||||||||||||||||||||||||
CG37    1      CTTCTTCCCTTCTTCTCCTCTCTAGGATTCTCTTTTCCCTTCCTTCCATCAATTCCCACT   60

CG104   61     TGGCTTTCTTCCCTTCTCTTGATCGACCTCACTCTACCCTCCATCTCGATTTTTGTTTCT   120
               ||||||||||||||||||||||||||||||||||||||||||||||.|||||||||||||
CG37    61     TGGCTTTCTTCCCTTCTCTTGATCGACCTCACTCTACCCTCCATCTTGATTTTTGTTTCT   120

CG104   121    AACTCTGTTACAGTTGCAACCCCCTTTCTTCTTCCTTTTTAATCTATTTTTATCCAATTT   180
               ||||||||||||||||||||||||||||||||||||||||||||||||||||||||||||
CG37    121    AACTCTGTTACAGTTGCAACCCCCTTTCTTCTTCCTTTTTAATCTATTTTTATCCAATTT   180

CG104   181    TTGTTCATGGGTTTTGCGTCTGGTGGTATATGAAGTGGTCAAAGCACGGTGAAGAAGCTC   240
               ||||||||||||||||||||||||||||||||||||||||||||||||||||||||||||
CG37    181    TTGTTCATGGGTTTTGCGTCTGGTGGTATATGAAGTGGTCAAAGCACGGTGAAGAAGCTC   240

CG104   241    CTAGAGAACCCCTTTGAATTTGGTGAATGGTAGGTTTCTGGAGCTGTGGAATTTTGTTGC   300
               ||||||||||||||||||||||||||||||||||||||||||||||||.|||||||||||
CG37    241    CTAGAGAACCCCTTTGAATTTGGTGAATGGTAGGTTTCTGGAGCTGTGCAATTTTGTTGC   300

CG104   301    CATTTATAAGGGGCGAATTCCCGAGAACGGAAGGGTTGTTGGGAAATTTTTGATATGGGT   360
               ||||||||||||||||||||||||||||||||||||||||||||||||||||||||||||
CG37    301    CATTTATAAGGGGCGAATTCCCGAGAACGGAAGGGTTGTTGGGAAATTTTTGATATGGGT   360

CG104   361    ATGGAGATTTTGGAGACGTATTCGTCTCTTCTACCTTCTGCAATGTTCTGTTTCTGAGAA   420
               ||||||||||||||||||||||||||||||||||||||||||||||||||||||||||||
CG37    361    ATGGAGATTTTGGAGACGTATTCGTCTCTTCTACCTTCTGCAATGTTCTGTTTCTGAGAA   420

CG104   421    TTTTGGAATTCAATTTCTGGATTTTGCTGTTGTTAGATGGTCGACGGGAAGGAGTTATTT   480
               ||||||||||||||||||||||||||||||||||||||||||||||||||||||||||||
CG37    421    TTTTGGAATTCAATTTCTGGATTTTGCTGTTGTTAGATGGTCGACGGGAAGGAGTTATTT   480
```

```
CG104   481   ATGTTCTACAAGGTAAGAATCTTTTAGGGTTGGGTTGGGTTGTGAATACACTTGATATTC   540
              |||||||||||||||||||||||||||||||||.||||||||||||||||||||||||||
CG37    481   ATGTTCTACAAGGTAAGAATCTTTTAGGGTTGGCTTGGGTTGTGAATACACTTGATATTC   540

CG104   541   AGttttttttttttttttttGAAATGTTAGTTTTTCGTGGTTGGCAGAGGAATTAATTGG   600
              ||||||||||||||||||||||||||||||||||||||||||||||||||||||||||||
CG37    541   AGTTTTTTTTTTTTTTTTTTGAAATGTTAGTTTTTCGTGGTTGGCAGAGGAATTAATTGG   600

CG104   601   CTTTGATTGAGATGATTACGTTTATGGATTCGAAAGAGAAACTGAAAGAGGTGGAGAAAT   660
              ||||||||||||||||||||||||||||||||||||||||||||||||||||||||||||
CG37    601   CTTTGATTGAGATGATTACGTTTATGGATTCGAAAGAGAAACTGAAAGAGGTGGAGAAAT   660

CG104   661   GTTTAGATCCTCAGTTATGGCATGCCTGTGCTGGAGGAATGGTTCAAATGCCGCCGGTGA   720
              ||||||||||||||||||||||||||||||||||||||||||||||||||||||||||||
CG37    661   GTTTAGATCCTCAGTTATGGCATGCCTGTGCTGGAGGAATGGTTCAAATGCCGCCGGTGA   720

CG104   721   ACGCCAGAGTTTTCTATTTTCCACAAGGTCATGCTGAGCATTCTTGTGCGCCAGTTGATT   780
              ||||||||||||||||||||||||||||||||||||||||||||||||||||||||||||
CG37    721   ACGCCAGAGTTTTCTATTTTCCACAAGGTCATGCTGAGCATTCTTGTGCGCCAGTTGATT   780

CG104   781   TCAGGAACTGTCCTAAGGTTCCTTCATATACCCTTTGCAGAGTTTCTGCCATCAAGTTCC   840
              ||||||||||||||||||||||||||||||||||||||||||||||||||||||||||||
CG37    781   TCAGGAACTGTCCTAAGGTTCCTTCATATACCCTTTGCAGAGTTTCTGCCATCAAGTTCC   840

CG104   841   TTGCAGATCCTGACACTGATGAGGTATTTGCCAAACTTAGGTTGATTCCCATAAATGGAA   900
              ||||||||||||||||||||||||||||||||||||||||||||||||||||||||||||
CG37    841   TTGCAGATCCTGACACTGATGAGGTATTTGCCAAACTTAGGTTGATTCCCATAAATGGAA   900

CG104   901   GCGAACTAGATTTTGAAGATGATGGAATTGGAAGGCTTAATGGGTCCGAACAGGATAAAC   960
              ||||||||||||||||||||||||||||||||||||||||||||||||||||||||||||
CG37    901   GCGAACTAGATTTTGAAGATGATGGAATTGGAAGGCTTAATGGGTCCGAACAGGATAAAC   960

CG104   961   CAACCTCATTTGCAAAGACACTGACTCAGTCTGATGCTAACAATGGTGGGGGTTTCTCTG   1020
              ||||||||||||||||||||||||||||||||||||||||||||||||||||||||||||
CG37    961   CAACCTCATTTGCAAAGACACTGACTCAGTCTGATGCTAACAATGGTGGGGGTTTCTCTG   1020

CG104   1021  TTCCAAGGTATTGTGCAGAAACTATCTTCCCTCGGTTGGATTATTCTGCTGATCCACCCG   1080
              ||||||||||||||||||||||||||||||||||||||||||||||||||||||||||||
CG37    1021  TTCCAAGGTATTGTGCAGAAACTATCTTCCCTCGGTTGGATTATTCTGCTGATCCACCCG   1080

CG104   1081  TTCAAACCATTCTTGCTAAGGATGTTCATGGGGAGACATGGAAATTCAGGCACATTTATA   1140
              ||||||||||||||||||||||||||||||||||||||||||||||||||||||||||||
CG37    1081  TTCAAACCATTCTTGCTAAGGATGTTCATGGGGAGACATGGAAATTCAGGCACATTTATA   1140

CG104   1141  GAGGGACGCCTCGCCGGCATCTTTTGACTACTGGCTGGAGTACTTTTGTTAACCATAAGA   1200
              ||||||||||||||||||||||||||||||||||||||||||||||||||||||||||||
CG37    1141  GAGGGACGCCTCGCCGGCATCTTTTGACTACTGGCTGGAGTACTTTTGTTAACCATAAGA   1200

CG104   1201  AGCTCGTTGCAGGTGATTCCATTGTTTTCCTAAGGGCAGAAAACGGAGATCTCTGCGTTG   1260
              ||||||||||||||||||||||||||||||||||||||||||||||||||||||||||||
CG37    1201  AGCTCGTTGCAGGTGATTCCATTGTTTTCCTAAGGGCAGAAAACGGAGATCTCTGCGTTG   1260

CG104   1261  GGATTAGACGGGCCAAGAGAGGAATTGGGGATGGACCGGAGTCATCCTGTGGCTGGAATC   1320
              ||||||||||||||||||||||||||||||||||||||||||||||||||||||||||||
CG37    1261  GGATTAGACGGGCCAAGAGAGGAATTGGGGATGGACCGGAGTCATCCTGTGGCTGGAATC   1320

CG104   1321  CAGCAGGTGGTAACTGTGCTGTTTCGTATGGGGCATTCTCTGCGTTTTTGAGGGAAGATG   1380
              ||||||||||||||||||||||||||||||||||||||||||||||||||||||||||||
CG37    1321  CAGCAGGTGGTAACTGTGCTGTTTCGTATGGGGCATTCTCTGCGTTTTTGAGGGAAGATG   1380

CG104   1381  ACAACAGATTGACGAGGTCTGCTAATGGCATGAATGGAAATGGAAGTCTTATGGGAAAGG   1440
              ||||||||||||||||||||||||||||||||||||||||||||||||||||||||||||
CG37    1381  ACAACAGATTGACGAGGTCTGCTAATGGCATGAATGGAAATGGAAGTCTTATGGGAAAGG   1440

CG104   1441  GGAAAGTGAAGCCTGAATCAGTTACTGAAGCTGCTAAACTTGCTTCAAATGGACAACCCT   1500
              ||||||||||||||||||||||||||||||||||||||||||||||||||||||||||||
CG37    1441  GGAAAGTGAAGCCTGAATCAGTTACTGAAGCTGCTAAACTTGCTTCAAATGGACAACCCT   1500
```

```
CG104  1501  TTGAAATAATATTCTATCCAAGAGCTAGTACTCCTGAATTCTGTGTCAAGGCAGCACTGG  1560
             ||||||||||||||||||||||||||||||||||||||||||||||||||||||||||||
CG37   1501  TTGAAATAATATTCTATCCAAGAGCTAGTACTCCTGAATTCTGTGTCAAGGCAGCACTGG  1560

CG104  1561  TGAAAGCAGCATTACAGATCCGGTGGTGCTCAGGTATGAGGTTCAAGATGGCCTTTGAAA  1620
             ||||||||||||||||||||||||||||||||||||||||||||||||||||||||||||
CG37   1561  TGAAAGCAGCATTACAGATCCGGTGGTGCTCAGGTATGAGGTTCAAGATGGCCTTTGAAA  1620

CG104  1621  CCGAGGACTCTTCACGAATTAGTTGGTTCATGGGTACCATCAATTCCGTTCAGGTTTCGG  1680
             ||||||||||||||||||||||||||||||||||||||||||||||||||||||||||||
CG37   1621  CCGAGGACTCTTCACGAATTAGTTGGTTCATGGGTACCATCAATTCCGTTCAGGTTTCGG  1680

CG104  1681  ACCCACTGCGCTGGCCTGAATCACCATGGAGGCTTCTCAGGTAATCACTGttttttttCT  1740
             ||||||||||||||||||||||||||||||||||||||||||||||||||||||||||||
CG37   1681  ACCCACTGCGCTGGCCTGAATCACCATGGAGGCTTCTCAGGTAATCACTGTTTTTTTTCT  1740

CG104  1741  CCCTGCCTGAATTCATTGCCAATAATCAATGTATTCTGAACATGGTGATTCAGGACCGAA  1800
             ||||||||||||||||||||||||||||||||||||||||||||||||||||||||||||
CG37   1741  CCCTGCCTGAATTCATTGCCAATAATCAATGTATTCTGAACATGGTGATTCAGGACCGAA  1800

CG104  1801  TATATCACGTCTGCAGAAACATAGAAGTTTGTAAGTTCAGTGATTGTCGGGATGATTTGT  1860
             ||||||||||||||||||||||||||||||||||||||||||||||||||||||||||||
CG37   1801  TATATCACGTCTGCAGAAACATAGAAGTTTGTAAGTTCAGTGATTGTCGGGATGATTTGT  1860

CG104  1861  AGTAGTTGACCTTACCACAACATTTCATTCAGTTTGTGCACTATTGATGGTTCTATGGAT  1920
             ||||||||||||||||||||||||||||||.|||||||||||||||||||||||||||||
CG37   1861  AGTAGTTGACCTTACCACAACATTTCATTCTGTTTGTGCACTATTGATGGTTCTATGGAT  1920

CG104  1921  TCATGGAAGGTCGTTCGTTGCCTTATCACTTGCTAATAACATATTTTGTGCAGGTTACTT  1980
             |||.||||||||||||||||||||||||||||||||||||||||||||||||||||||||
CG37   1921  TCACGGAAGGTCGTTCGTTGCCTTATCACTTGCTAATAACATATTTTGTGCAGGTTACTT  1980

CG104  1981  GGGATGAACCAGATTTACTTCAGAATGTGAAACGCGTTAGCCCATGGTTGGTTGAATTGG  2040
             ||||||||||||||||||||||||||||||||||||||||||||||||||||||||||||
CG37   1981  GGGATGAACCAGATTTACTTCAGAATGTGAAACGCGTTAGCCCATGGTTGGTTGAATTGG  2040


CG104  2041  TATCAAGCATGTCTCCAATTCACCTTGCCCCTTTCTCACCACCAAGGAAAAAGTTCAGAT  2100
             ||||||||||||||||||||||||||||||||||||||||||||||||||||||||||||
CG37   2041  TATCAAGCATGTCTCCAATTCACCTTGCCCCTTTCTCACCACCAAGGAAAAAGTTCAGAT  2100

CG104  2101  ATCCACAACACCCTGATTTCCCCCTTGATAATCAACCTCCTGTGCCATCGTTTTCTAGTT  2160
             ||||||||||||||||||||||||||||||||||||||||||||||||||||||||||||
CG37   2101  ATCCACAACACCCTGATTTCCCCCTTGATAATCAACCTCCTGTGCCATCGTTTTCTAGTT  2160

CG104  2161  ATCTCCATGGGACTGGCAGCCCCTTCGGTTGTCCTCCCGACAACAACCCTGCTGGCATGC  2220
             ||||||||||||||||||||||||||||||||||||||||||||||||||||||||||||
CG37   2161  ATCTCCATGGGACTGGCAGCCCCTTCGGTTGTCCTCCCGACAACAACCCTGCTGGCATGC  2220

CG104  2221  AGGGAGCCAGGCATGCTCATTTTGGTCTATCCTTGTCAGATTTTCATGTCAGTAAACTGC  2280
             ||||||||||||||||||||||||||||||||||||||||||||||||||||||||||||
CG37   2221  AGGGAGCCAGGCATGCTCATTTTGGTCTATCCTTGTCAGATTTTCATGTCAGTAAACTGC  2280

CG104  2281  AGTCAGGTCTGTTTTCGATTGGTTATCGATCACTGGATCCAGCTGCTGGATCAACTAGAC  2340
             ||||||||||||||||||||||||||||||||||||||||||||||||||||||||||||
CG37   2281  AGTCAGGTCTGTTTTCGATTGGTTATCGATCACTGGATCCAGCTGCTGGATCAACTAGAC  2340

CG104  2341  TTTCTGGTAATGTAATGACTGAAAAACCAAGTATGAGTGAAAACGTATCTTGCTTGCTAA  2400
             ||||||||||||||||||||||||||||||||||||||||||||||||||||||||||||
CG37   2341  TTTCTGGTAATGTAATGACTGAAAAACCAAGTATGAGTGAAAACGTATCTTGCTTGCTAA  2400

CG104  2401  CCATGGCACATTCTACTCAAGCTTCAAAGAAATTTGATGGCGTGAAGACTCCTCAGCTAA  2460
             ||||||||||||||||||||||||||||||||||||||||||||||||||||||||||||
CG37   2401  CCATGGCACATTCTACTCAAGCTTCAAAGAAATTTGATGGCGTGAAGACTCCTCAGCTAA  2460

CG104  2461  TACTTTTTGGCCGACCCATACTTACAGAATTGCAGATGTCTCAAAGCTTCTCTGGCGATA  2520
             ||||||||||||||||||||||||||||||||||||||||||||||||||||||||||||
```

```
CG37   2461  TACTTTTTGGCCGACCCATACTTACAGAATTGCAGATGTCTCAAAGCTTCTCTGGCGATA  2520

CG104  2521  CCGTTTCTCCAGTAGGTACTGGAAATAGTTCGTCAGATGGAAATGGAGACAAAATGACAA  2580
             ||||||||||||||||||||||||||||||||||||||||||||||||||||||||||||
CG37   2521  CCGTTTCTCCAGTAGGTACTGGAAATAGTTCGTCAGATGGAAATGGAGACAAAATGACAA  2580

CG104  2581  ACCTCTCCGATGGCTCTGGATCTGCTTTACATCAACAAGGTCTACCAGAAGGCTCTGCTG  2640
             |||||||||||||||||||||||||||||||||||||||||||||||||||||||.||||
CG37   2581  ACCTCTCCGATGGCTCTGGATCTGCTTTACATCAACAAGGTCTACCAGAAGGCTCGGCTG  2640

CG104  2641  GCGAAAATTTCCAGTGGTACAAGGACAATTGCCAAGAAATCGACCCAAACTTGGATATCG  2700
             |||||||||||||||||||||||||||||.||||||||||||||||||||||||||||||
CG37   2641  GCGAAAATTTCCAGTGGTACAAGGACAATCGCCAAGAAATCGACCCAAACTTGGATATCG  2700

CG104  2701  GCCACTGTAAAGTCTTTATGGAATCAGAAGATGTAGGACGCACTCTCGATCTTTCTTCAC  2760
             ||||||||||||||||||||||||||||||||||||||||||||||||||||||||||||
CG37   2701  GCCACTGTAAAGTCTTTATGGAATCAGAAGATGTAGGACGCACTCTCGATCTTTCTTCAC  2760

CG104  2761  TTGGGTCTTATGAAGAATTGTACAGAAAACTTGGAAATATGTTTGGTATAGATAATTCAG  2820
             ||||||||||||||||||||||||||||||||||||||||||||||||||||||||||||
CG37   2761  TTGGGTCTTATGAAGAATTGTACAGAAAACTTGGAAATATGTTTGGTATAGATAATTCAG  2820

CG104  2821  AGACATTGAACCATGTCTTGTACCGTGATGTTTCCGGTGCTGTCAAACATGTCGGTGACG  2880
             ||||||||||||||||||||||||||||||||||||||||||||||||||||||||||||
CG37   2821  AGACATTGAACCATGTCTTGTACCGTGATGTTTCCGGTGCTGTCAAACATGTCGGTGACG  2880

CG104  2881  AACAATTCAGGTATATTTCTTCTTCCTTTGGCAACGTTGAGACATCGTCTTACATTCTGT  2940
             ||||||||||||||||||||||||||||||||||||||||||||||||||||||||||||
CG37   2881  AACAATTCAGGTATATTTCTTCTTCCTTTGGCAACGTTGAGACATCGTCTTACATTCTGT  2940

CG104  2941  CATTTCCAATACATATCACGTCTGATTAACGCTCCTCGTTTTCTGTTTCAGTGACTTCAT  3000
             |||||||||||||||||||||||||.||||||||||||||||||||||||||||||||||
CG37   2941  CATTTCCAATACATATCACGTCTGAGTAACGCTCCTCGTTTTCTGTTTCAGTGACTTCAT  3000

CG104  3001  CAAGACAGCAAGAAGATTGACAATTCTAACAGATTCAGGAAGTAACAATGTAGGAGCTTA  3060
             ||||||||||||||||||||||||||||||||||||||||||||||||||||||||||||
CG37   3001  CAAGACAGCAAGAAGATTGACAATTCTAACAGATTCAGGAAGTAACAATGTAGGAGCTTA  3060

CG104  3061  GAAGGAAGAATAAGAGTTGATTTCAACTGTACAGCTGGTCAATATCAGGTCCTTCATTTC  3120
             ||||||||||||||||||||||||||||||||||||||||||||||||||||||||||||
CG37   3061  GAAGGAAGAATAAGAGTTGATTTCAACTGTACAGCTGGTCAATATCAGGTCCTTCATTTC  3120

CG104  3121  CTTGCTGACCGTTCTCTGTTTCAAATCTGGGAGCTGCTGCTAAGATTCAAGCtttttttA  3180
             ||||||||||||||||||||||||||||||||||||||||||||||||||||||||||||
CG37   3121  CTTGCTGACCGTTCTCTGTTTCAAATCTGGGAGCTGCTGCTAAGATTCAAGCTTTTTTTA  3180

CG104  3181  ACTATGAAAAAAGCTGATATTTTTCCTTTTCCTTATAATTAAtttttttttttCCCAATC  3240
             ||||||||||||||||||||||||||||||||||||||||||||||||||||||||||||
CG37   3181  ACTATGAAAAAAGCTGATATTTTTCCTTTTCCTTATAATTAATTTTTTTTTTTCCCAATC  3240

CG104  3241  CAACTTCTGGAGGGAATGTTTGAATTTTGGAACTTGCTTACATCCCCATGTTCTGTGATG  3300
             ||||||||||||||||||||||||||||||||||||||||||||||||||||||||||||
CG37   3241  CAACTTCTGGAGGGAATGTTTGAATTTTGGAACTTGCTTACATCCCCATGTTCTGTGATG  3300

CG104  3301  AACTTTGAGGGGGtttctttttGGGGGttttttttCTTCTTTCCATTTTAGGTGGTTAGT  3360
             ||||||||||||||||||||||||||||||||||||||||||||||||||||||||||||
CG37   3301  AACTTTGAGGGGGTTTCTTTTTGGGGGTTTTTTTTCTTCTTTCCATTTTAGGTGGTTAGT  3360

CG104  3361  TCTTATTATATAAGTGGAAGTGATTTGAGAGAGTATTGGGAATGTGTTTGTAAGAGAAGC  3420
             ||||||||||||||||||||||||||||||||||||||||||||||||||||||||||||
CG37   3361  TCTTATTATATAAGTGGAAGTGATTTGAGAGAGTATTGGGAATGTGTTTGTAAGAGAAGC  3420

CG104  3421  ATTGTGAAATATAATGAGTTAAAGTGGAGTTGTTTCTAAAGGTTTGTGGTCAAAGTAAGT  3480
             ||||||||||||||||||||||||||||||||||||||||||||||||||||||||||||
CG37   3421  ATTGTGAAATATAATGAGTTAAAGTGGAGTTGTTTCTAAAGGTTTGTGGTCAAAGTAAGT  3480

CG104  3481  CATAAAAGAAAGGCATTTTGAAGAAGAAAAGACAAAAAGGGCTTACCTTGAAACTTGAGT  3540
             ||||||||||||||||||||||||||||||||||||||||||||||||||||||||||||
```

```
CG37    3481  CATAAAAGAAAGGCATTTTGAAGAAGAAAAGACAAAAAGGGCTTACCTTGAAACTTGAGT  3540

CG104   3541  TTCATTTGTAAAAGTTCAATGTTAATGATGTAGTGAGTTGTGAGTTTGTTGAATTAATGC  3600
              ||||||||||||||||||||||||||||||||||||||||||||||||||||||||||||
CG37    3541  TTCATTTGTAAAAGTTCAATGTTAATGATGTAGTGAGTTGTGAGTTTGTTGAATTAATGC  3600

CG104   3601  CATTCCCCAAAAGATGGGGAATTCAAAAGTTTCATAAAAAAGAAAGTGGTTTTCATTCAT  3660
              ||||||||||||||||||||||||||||||||||||||||||||||||||||||||||||
CG37    3601  CATTCCCCAAAAGATGGGGAATTCAAAAGTTTCATAAAAAAGAAAGTGGTTTTCATTCAT  3660

CG104   3661  TGTATGACCAAAATGAATGATGTAAATTTGTTTGGTTGGAAATCTACTCTACTATTAGTC  3720
              ||||||||||||||||||||||||||||||||||||||||||||||||||||||||||||
CG37    3661  TGTATGACCAAAATGAATGATGTAAATTTGTTTGGTTGGAAATCTACTCTACTATTAGTC  3720

CG104   3721  T  3721
              |
CG37    3721  T  3721
```

Csa6G445230

```
CG104   1     TTCTCTTTCTCTTTCTCTTTCTATATATAAAGCTTATGCAATTAACTCTCTGGCGTTACT  60
              ||||||||||||||||||||||||||||||||||||||||||||||||||||||||||||
CG37    1     TTCTCTTTCTCTTTCTCTTTCTATATATAAAGCTTATGCAATTAACTCTCTGGCGTTACT  60

CG104   61    GAAGCATCTATGAGATCTACTGTCCACACTGAATTCATGGATTCAATGAGATTTCCTACG  120
              ||||||||||||||||||||||||||||||||||||||||||||||||||||||||||||
CG37    61    GAAGCATCTATGAGATCTACTGTCCACACTGAATTCATGGATTCAATGAGATTTCCTACG  120

CG104   121   AATTAAGCCAGTACGGAAAACCCAGGTGAATATCAAAAGAAGTAGAACAGCAATGAAATC  180
              ||||||||||.||||||||||||||||||||||||||||||||||||||||||.||||||
CG37    121   AATTAAGCCAATACGGAAAACCCAGGTGAATATCAAAAGAAGTAGAACAGCAACGAAATC  180

CG104   181   AACAAACAATTTTACAACCCTATCTCCTTTTTTTTTCTTTTCTTTTTTGTTCAGCTTCAT  240
              ||||||||||||||||||||||||||||||||||||||||||||||||||||||||||||
CG37    181   AACAAACAATTTTACAACCCTATCTCCTTTTTTTTTCTTTTCTTTTTTGTTCAGCTTCAT  240

CG104   241   GTGGGTTTCTTTTGTTTATCCATAACCTCTCTTAAAATCTGTGTTTTTGAAGCTTGGGTT  300
              ||||||||||||||||||||||||||||||||||||||||||||||||||||||||||||
CG37    241   GTGGGTTTCTTTTGTTTATCCATAACCTCTCTTAAAATCTGTGTTTTTGAAGCTTGGGTT  300

CG104   301   TTAATTCCTTTTCTTTTCTTCACCTTTAGCTTGCATCGGATCCGTGGTTTTTTATTCTGA  360
              ||||||||||||||||||||||||||||||||||||||||||||||||||||||||||||
CG37    301   TTAATTCCTTTTCTTTTCTTCACCTTTAGCTTGCATCGGATCCGTGGTTTTTTATTCTGA  360

CG104   361   TCTTCATTTCTCGTGGCCAATTTCTTTTGGGAGAGTAGGTTTTGGGTTCTGGGTTCTGGG  420
              ||||||||||||||||||||||||||||||||||||||||||||||||||||||||||||
CG37    361   TCTTCATTTCTCGTGGCCAATTTCTTTTGGGAGAGTAGGTTTTGGGTTCTGGGTTCTGGG  420

CG104   421   TTGGGCTTTATTTTAGGTGTATGTCCCAATTTTGAGATACCCTTTTGTGTTCGATGAAAG  480
              ||||||||||||||||||||||||||||||||||||||||||||||||||||||||||||
CG37    421   TTGGGCTTTATTTTAGGTGTATGTCCCAATTTTGAGATACCCTTTTGTGTTCGATGAAAG  480

CG104   481   TCCCCGGTATTTTTTTTATTTTTTATTTTTTTTTTTTATAATTTTGAGCTGTTTTTCTGG  540
              ||||||||||||||||||||||||||||||||||||||||||||||||||||||||||||
CG37    481   TCCCCGGTATTTTTTTTATTTTTTATTTTTTTTTTTTATAATTTTGAGCTGTTTTTCTGG  540

CG104   541   ATTTTGACTGTCTAGGGTTTGAATTGTAATGGGGCTCTATTAATTTTGAACTATAGGTAT  600
              ||||||||||||||||||||||||||||||||||||||||||||||||||||||||||||
CG37    541   ATTTTGACTGTCTAGGGTTTGAATTGTAATGGGGCTCTATTAATTTTGAACTATAGGTAT  600

CG104   601   GCATTTAGGTTGTTGACTTTCTATAAGGAGAAATAAAAAATAGGCGGTAAGGTAAGGCCG  660
              ||||||||||||||||||||||||||||||||||||||||||||||||||||||||||||
CG37    601   GCATTTAGGTTGTTGACTTTCTATAAGGAGAAATAAAAAATAGGCGGTAAGGTAAGGCCG  660

CG104   661   AACCAGGATTATTTATTATTAGAATTACTCATGGTAGTTAACATGTAGAATTCTTGTACC  720
              ||||||||||||||||||||||||||||||||||||||||||||||||||||||||||||
CG37    661   AACCAGGATTATTTATTATTAGAATTACTCATGGTAGTTAACATGTAGAATTCTTGTACC  720
```

```
CG104   721    TCCCTAAATCGAGTTTTCTTAATTGCTAAACTGATTTCGTGGGGAATTAGCTGCTTTTTC   780
               |||||||||||||||||||||||||||||||||||||||||||.||||||||||||||||
CG37    721    TCCCTAAATCGAGTTTTCTTAATTGCTAAACTGATTTCGTGGGAAATTAGCTGCTTTTTC   780

CG104   781    AGTTTGTTTAGTCAACATCTGATTTTGATTCCTAAAAATTTATCCTATGCATGATTTCAC   840
               ||||||||||||||||||||||||||||||||||||||||||||||||||||||||||||
CG37    781    AGTTTGTTTAGTCAACATCTGATTTTGATTCCTAAAAATTTATCCTATGCATGATTTCAC   840

CG104   841    AATTTTGTTGTCTTTATAGTAAAGTTTTGATTCTGGACTTTCGGTTTCTACTTTATTATT   900
               ||||||||||||||||||||||||||||||||||||||||||||||||||||||||||||
CG37    841    AATTTTGTTGTCTTTATAGTAAAGTTTTGATTCTGGACTTTCGGTTTCTACTTTATTATT   900

CG104   901    GTAATTTTGAGATCCTATCATCACCTATTGTTTCTCATAGATTTCTTTTACCAAAAATCT   960
               ||.|||||||||||||||||||||||||||||||||||||||||||||||||||||||||
CG37    901    GTGATTTTGAGATCCTATCATCACCTATTGTTTCTCATAGATTTCTTTTACCAAAAATCT   960

CG104   961    ATTATTTCTTTGAATGTATTGCATAAACAACTATTTTGTTCTGCATTACCGTTGTCTCCT   1020
               |||||||||||||||||||||||||||||||||||||||||.||||||||||||||||||
CG37    961    ATTATTTCTTTGAATGTATTGCATAAACAACTATTTTGTTCCGCATTACCGTTGTCTCCT   1020


CG104   1021   TCTCTGATGCTAATGGATATGCTTTCATTGATTTAGGCGAGGGGTGGCACAGAATATTCT   1080
               ||||||||||||||||||.|.|||||||||||||||||||||||||||||||||||||||
CG37    1021   TCTCTGATGCTAATGGATGTCCTTTCATTGATTTAGGCGAGGGGTGGCACAGAATATTCT   1080

CG104   1081   TTTCGGAGCAGGCACTAAATTAATAAGTTATCAAGATCACTCTAGAGTGCCATTCAATTG   1140
               ||||||||||||||||||||||||||||||||||||||||||||||||||||||||||||
CG37    1081   TTTCGGAGCAGGCACTAAATTAATAAGTTATCAAGATCACTCTAGAGTGCCATTCAATTG   1140

CG104   1141   GCATTCATTCTAAGAATGCTAAGGAAGATAACCACAGCTATCGTTCGAGGATTTTCGTGG   1200
               ||||||||||||||||||||||||||||||||||||||||||||||||||||||||||||
CG37    1141   GCATTCATTCTAAGAATGCTAAGGAAGATAACCACAGCTATCGTTCGAGGATTTTCGTGG   1200

CG104   1201   CGTTAGGACTGAGGGATTGAGCACATATAATGTGCTAGAAGGATATTTGTTGAAGCAAGG   1260
               ||||||||||||||||||||||||||||||||||||||||||||||||||||||||||||
CG37    1201   CGTTAGGACTGAGGGATTGAGCACATATAATGTGCTAGAAGGATATTTGTTGAAGCAAGG   1260

CG104   1261   ATGGATGTTCATGTTATATTTGCACAATATAATGGAACAGGGGTTATTTACAATGGATGA   1320
               |||||||||||||||||||||||||||.||||||||||||||||||||||||||||||||
CG37    1261   ATGGATGTTCATGTTATATTTGCACAACATAATGGAACAGGGGTTATTTACAATGGATGA   1320

CG104   1321   TTTTTGTTCTTTTTTCCTTTTTTCTTCGGTTGTAGAAATTTTTACTTTCTAGTTAGTAAA   1380
               ||||||||||||||||||||||||||||||||||||||||||||||||||||||||||||
CG37    1321   TTTTTGTTCTTTTTTCCTTTTTTCTTCGGTTGTAGAAATTTTTACTTTCTAGTTAGTAAA   1380

CG104   1381   AGAGTGGTAGTTGCAGAGAACACAAACACCTGATGTAAATTGTTTATCTCGATGCTTATT   1440
               ||||||||||||||||||||||||||||||||||||||||||||||||||||||||||||
CG37    1381   AGAGTGGTAGTTGCAGAGAACACAAACACCTGATGTAAATTGTTTATCTCGATGCTTATT   1440

CG104   1441   AGGAAGTTTGTAGTTTTCTGGACTTGTTAGCCACGCTTTACACACTGCTCCATTCCGACT   1500
               ||||||||||||||||||||||||||||||||||||||||||||||||||||||||||||
CG37    1441   AGGAAGTTTGTAGTTTTCTGGACTTGTTAGCCACGCTTTACACACTGCTCCATTCCGACT   1500

CG104   1501   ATTTGTGGAAAATCTCCATTTATACCATGGAATCTACGACATTGCATACAACTCATCAG   1560
               ||||||||||||||||||||||||||||||||||||||||||||||||||||||||||||
CG37    1501   ATTTGTGGAAAATCTCCATTTATACCATGGAATCTACGACATTGCATACAACTCATCAG   1560

CG104   1561   TCGGGTGCTATTCATAGGTTTATACCTTTCATTGCACCTGCACTTCTAGTTTCAATTAGT   1620
               ||||||||||||||||||||||||||||||||||||||||||||||||||||||||||||
CG37    1561   TCGGGTGCTATTCATAGGTTTATACCTTTCATTGCACCTGCACTTCTAGTTTCAATTAGT   1620

CG104   1621   TATGTTGACCCTGGAAAGTGGGCTGCAACTGTTGAAGGAGGTGCTCGGTTTGGCTTTGAT   1680
               ||||||||||||||||||||||||||||||||||||||||||||||||||||||||||||
CG37    1621   TATGTTGACCCTGGAAAGTGGGCTGCAACTGTTGAAGGAGGTGCTCGGTTTGGCTTTGAT   1680

CG104   1681   TTGTTTGTGTTAGTGCTTCTTTTCAATCTTGCTGCTATTTTATGCCAGTATCTCTCAGCT   1740
               ||||||||||||||||||||||||||||||||||||||||||||||||||||||||||||
```

```
CG37   1681  TTGTTTGTGTTAGTGCTTCTTTTCAATCTTGCTGCTATTTTATGCCAGTATCTCTCAGCT  1740

CG104  1741  AGCATTGGTGTGGTCACTGGAAGAGGTCTTGCCCAGGTAATTTATTTGTTTTCAAGGCTT  1800
             ||||||||||||||||||||||||||||||||||||||||||||.|||||||||||||||
CG37   1741  AGCATTGGTGTGGTCACTGGAAGAGGTCTTGCCCAGGTAATTTAATTGTTTTCAAGGCTT  1800

CG104  1801  TCGCATTTTTACTTATGTTGTGTCTATCAAGCAGCTTTTATCTGAAAATCAGTCAATTGA  1860
             |.||||||||||||||||||||||||||||||||||||||||||||||||||||||||||
CG37   1801  TAGCATTTTTACTTATGTTGTGTCTATCAAGCAGCTTTTATCTGAAAATCAGTCAATTGA  1860

CG104  1861  CCGATGTTGATTTTTCCCTTTATTAGTTTTGAATTTTTTTTTATGGGAGAATTTCATGT  1920
             |||||||||||||||||||||||||||||||||..|||||||||||||||||||||||||
CG37   1861  CCGATGTTGATTTTTCCCTTTATTAGTTTTGAAAATTTTTTTATGGGAGAATTTCATGT  1920

CG104  1921  GTTGGTTGTATCTTGTATTCTCCTATGTGGTTCGTTTGCATCTTTCTCATTAGTCTTCAA  1980
             ||||||||||||||||||||||||||||||||.||||||.||||||||||||||||||||
CG37   1921  GTTGGTTGTATCTTGTATTCTCCTATGTGGTTTGTTTGCGTCTTTCTCATTAGTCTTCAA  1980

CG104  1981  TTTCGAGGACTTTTTTGTAATTATCTTTTAGACACGAATCTCTATGGTTGAATTCCCTTC  2040
             |||||||||||||||||||||||||||||||||||||||||||.||||||||||||||||
CG37   1981  TTTCGAGGACTTTTTTGTAATTATCTTTTAGACACGAATCTCTGTGGTTGAATTCCCTTC  2040

CG104  2041  TTGTAGAAGGGGTCCCCTGTTTTTTTGTTGGCTTTG-TTTTTTTATGCCCGTTTATTCTT  2099
             |||||||||||||||||||||||||||||||||||| |||||||||||||||||||||||
CG37   2041  TTGTAGAAGGGGTCCCCTGTTTTTTTGTTGGCTTTGTTTTTTTTATGCCCGTTTATTCTT  2100

CG104  2100  TCATTTATTCTCAATGAAAGTTGTTCTTATAAATAAATAAATGTGTGTGGACGTGTCTCT  2159
             ||||||||||||||||||||||||||||||||||||||||||||||||||||||||||||
CG37   2101  TCATTTATTCTCAATGAAAGTTGTTCTTATAAATAAATAAATGTGTGTGGACGTGTCTCT  2160

CG104  2160  GTTTCCAGTTTGGGTTGATGTTTGATATGTTAGCTTGTAACCTTCACTTTCACTTTATGC  2219
             ||||||||||||||||||||||||||||||||||||||||||||||||||||||||||||
CG37   2161  GTTTCCAGTTTGGGTTGATGTTTGATATGTTAGCTTGTAACCTTCACTTTCACTTTATGC  2220

CG104  2220  CTCTTCCATTTTGGTTTGTTCCATGGGTATAGGTTGATTTTTGTTATTTTTTAAATTACT  2279
             ||||||||||||||||||||||||||||||||||||||||||||||||||||||||||||
CG37   2221  CTCTTCCATTTTGGTTTGTTCCATGGGTATAGGTTGATTTTTGTTATTTTTTAAATTACT  2280

CG104  2280  TGACTGCTAGTTGTTAAATGTTACTGGAGGTTGACATATTTACCCTATTTTGTTTTGTTG  2339
             ||||||||||||||||||||||||||||||||||||||||||||||||||||||||||||
CG37   2281  TGACTGCTAGTTGTTAAATGTTACTGGAGGTTGACATATTTACCCTATTTTGTTTTGTTG  2340

CG104  2340  ATTATACCAAAGATATGCAACGAGGAGTATGATAAGTGTACATGTTTCTTCCTGGGAATC  2399
             |||||||||||||||||||.||||||||||||||||||||||||||||||||||||||||
CG37   2341  ATTATACCAAAGATATGCAGCGAGGAGTATGATAAGTGTACATGTTTCTTCCTGGGAATC  2400

CG104  2400  CAAGCAGAGGCTTCTGTGATTCTGTTAGACCTTAACATGGTATTTTATTTTGTTTCTGCT  2459
             ||||||||||||||||||||||||||||||||||||||||||||||||||||||||||||
CG37   2401  CAAGCAGAGGCTTCTGTGATTCTGTTAGACCTTAACATGGTATTTTATTTTGTTTCTGCT  2460

CG104  2460  AAGTTATTCCAGACTTCCAGCCAAACCTGTGATTCAGTTTGTTAAGATTTTTTTTAGTG  2519
             ||||||||||||||||||||||||||||||||||||||||||||||||||||||||||||
CG37   2461  AAGTTATTCCAGACTTCCAGCCAAACCTGTGATTCAGTTTGTTAAGATTTTTTTTAGTG  2520

CG104  2520  AAGCATGGTCTAATTTGACCATATTTTAATTCTTCATTTTCATTATAAAGCTAACATGTC  2579
             ||||||||||||||||||||||||||||||||||||||||||||||||||||||||||||
CG37   2521  AAGCATGGTCTAATTTGACCATATTTTAATTCTTCATTTTCATTATAAAGCTAACATGTC  2580

CG104  2580  ATTTAAAAAACATACTAGATCTTGGGCATTTCAAATGGACTTAATCTTCTACTTGGGTGG  2639
             ||||||||||||||||||||||||||||||||||||||||||||||||||||||||||||
CG37   2581  ATTTAAAAAACATACTAGATCTTGGGCATTTCAAATGGACTTAATCTTCTACTTGGGTGG  2640

CG104  2640  GACCTCTTCACATGTGTCCTTTTGACGGGTGTTGCTGCTGCTTTATTTCCTCCTTTTGCT  2699
             ||||||||||||||||||||||||||||||||||||||||||||||||||||||||||||
CG37   2641  GACCTCTTCACATGTGTCCTTTTGACGGGTGTTGCTGCTGCTTTATTTCCTCCTTTTGCT  2700

CG104  2700  GACCTTCTGGTAAATAGAACTTTTAATCAATCATGCATAACTTTCTCTTTGATGTAATTC  2759
```

```
            |||||||||||||||||||||||||||||||||||||.||||||||||||||||||||||
CG37   2701 GACCTTCTGGTAAATAGAACTTTTAATCAATCATGCACAACTTTCTCTTTGATGTAATTC 2760

CG104  2760 ATTATCTCAAGGGTTTCTATTTTCTAGGAAGATGGCAGGGCAAAGTTCCTCTATATATGT 2819
            ||||||||||||||||||||||||||||||||||||||||||||||||||||||||||||
CG37   2761 ATTATCTCAAGGGTTTCTATTTTCTAGGAAGATGGCAGGGCAAAGTTCCTCTATATATGT 2820

CG104  2820 ATGGCGGGATTTGTACTGCTCTCTTTGGTTCTTGGAGTATTAATCAGTCAACCTGAAATC 2879
            ||||||||||||||||||||||||||||||||||||||||||||||||||||||||||||
CG37   2821 ATGGCGGGATTTGTACTGCTCTCTTTGGTTCTTGGAGTATTAATCAGTCAACCTGAAATC 2880

CG104  2880 CCACTTTCCATGAATCTCATGCCGACAAGGTTAAATGGGGAAAGTGCCTTTACTCTTATG 2939
            ||||||||||||||||||||||||||||||||||||||||||||||||||||||||||||
CG37   2881 CCACTTTCCATGAATCTCATGCCGACAAGGTTAAATGGGGAAAGTGCCTTTACTCTTATG 2940

CG104  2940 AGTCTTCTTGGAGCAAGTGTCATGCCACACAATTTTTATGTGCATTCTTCTATTGTGCAG 2999
            ||||||||||||||||||||||||||||||||||||||||||||||||||||||||||||
CG37   2941 AGTCTTCTTGGAGCAAGTGTCATGCCACACAATTTTTATGTGCATTCTTCTATTGTGCAG 3000

CG104  3000 GTACTTCTCGTTTCTTCTATCGCCATTTGTATTTGATTGAGCATAAAACTGCAAATGTTT 3059
            ||||||||||||||||||||||||||||||||||||||||||||||||||||||||||||
CG37   3001 GTACTTCTCGTTTCTTCTATCGCCATTTGTATTTGATTGAGCATAAAACTGCAAATGTTT 3060

CG104  3060 TACTTTTCTTAGTCAATGTGTATGTGCAGACAAGGAAAGTCCAATGAGATGATTTTTGCC 3119
            ||||||||||||||||||||||||||||||||||||||||||||||||||||||||||||
CG37   3061 TACTTTTCTTAGTCAATGTGTATGTGCAGACAAGGAAAGTCCAATGAGATGATTTTTGCC 3120

CG104  3120 TACCAATATCTTAAGATTAGGATGTCATTCTAGGCGGTTGATCAAGTTCAAAACTTCTGG 3179
            ||||||||||||||||||||||||||||||||||||||||||||||||||||||||||||
CG37   3121 TACCAATATCTTAAGATTAGGATGTCATTCTAGGCGGTTGATCAAGTTCAAAACTTCTGG 3180

CG104  3180 GATTCAAATGTTTTAATCATTTAGATTTCTTCATAGTATAAATTTGGATATCCTTATGTG 3239
            ||||||||||||||||||||||||||||||||||||||||||||||||||||||||||||
CG37   3181 GATTCAAATGTTTTAATCATTTAGATTTCTTCATAGTATAAATTTGGATATCCTTATGTG 3240

CG104  3240 CTTTATAATGATTTTCCTTTCTTCTTTTTGCAGCAGCACCAGAGTCCACCAAATATTTCC 3299
            ||||||||||||||||||||||||||||||||||||||||||||||||||||||||||||
CG37   3241 CTTTATAATGATTTTCCTTTCTTCTTTTTGCAGCAGCACCAGAGTCCACCAAATATTTCC 3300

CG104  3300 AAAGAAGTTTCGTGTTATAATCATTTGTTTGCTATTTTCTGCATATTCAGTGGAATTTAT 3359
            ||||||||||||||||||||||||||||||||||||||||||||||||||||||||||||
CG37   3301 AAAGAAGTTTCGTGTTATAATCATTTGTTTGCTATTTTCTGCATATTCAGTGGAATTTAT 3360

CG104  3360 GTGGTGAATAACGTTCTCATGAACTCAGCTGCAAATGTATTCTATAGCAGTGGTCTTGCT 3419
            ||||||||||||||||||||||||||||||||||||||||||||||||||||||||||||
CG37   3361 GTGGTGAATAACGTTCTCATGAACTCAGCTGCAAATGTATTCTATAGCAGTGGTCTTGCT 3420

CG104  3420 TTGCACACCTTTACAGATGCATTGTCTTTAATGGAGCAGGTGTTCTAGAGTCCCCCCCTC 3479
            ||||||||||||.|||||||||||||||||||||||||||||||||||||||||||||||
CG37   3421 TTGCACACCTTTTCAGATGCATTGTCTTTAATGGAGCAGGTGTTCTAGAGTCCCCCCCTC 3480

CG104  3480 CCTTTTAAATTTATTTATTTTTGTTTACTTGCTCCTTAACACAGTTTTACTGTAATAAGT 3539
            ||||||||||||||||||||||||||||||||||||||||||||||||||||||||||||
CG37   3481 CCTTTTAAATTTATTTATTTTTGTTTACTTGCTCCTTAACACAGTTTTACTGTAATAAGT 3540

CG104  3540 ATTGATTTTATTTTCCATTTCCCTCCAAACTATGCAGGTATTTGGGAGCTCAGTGGTATA 3599
            ||||||||||||||||||||||||||||||||||||||||||||||||||||||||||||
CG37   3541 ATTGATTTTATTTTCCATTTCCCTCCAAACTATGCAGGTATTTGGGAGCTCAGTGGTATA 3600

CG104  3600 TGTTCTCTTCTTACTTGTTTTGTTTCTATCAAATCAAATCACAGCTCTCACATGGAGTCT 3659
            ||||||||||||||||||||||||||||||||||||||||||||||||||||||||||||
CG37   3601 TGTTCTCTTCTTACTTGTTTTGTTTCTATCAAATCAAATCACAGCTCTCACATGGAGTCT 3660

CG104  3660 TGGTGGTCAACTGGTTCTGACCAATTTCTTAAAATTAGATATTCCTGGTTGGCTCCATTG 3719
            ||||||||||||||||||||||||||||||||||||||||||||||||||||||||||||
CG37   3661 TGGTGGTCAACTGGTTCTGACCAATTTCTTAAAATTAGATATTCCTGGTTGGCTCCATTG 3720
```

```
CG104  3720  TGCTACAATTAGGATTATTGCCATTATTCCAGCACTATGCTGTGTCTGGAGTTCGGGTGC  3779
             ||||||||||||||||||||||||||||||||||||||||||||||||||||||||||||
CG37   3721  TGCTACAATTAGGATTATTGCCATTATTCCAGCACTATGCTGTGTCTGGAGTTCGGGTGC  3780

CG104  3780  TGAAGGGATGTATCAACTTCTTATATTTTCTCAGGTTATGGTAGCTCTATTGCTTCCATC  3839
             ||||||||||||||||||||||||||||||||||||||||||||||||||||||||||||
CG37   3781  TGAAGGGATGTATCAACTTCTTATATTTTCTCAGGTTATGGTAGCTCTATTGCTTCCATC  3840

CG104  3840  TTCTGTGATTCCCCTCTATCGTGTTGCTTCATCAAGAACAATAATGGGTGCCCTCAAAAT  3899
             ||||||||||||||||||||||||||||||||||||||||||||||||||||||||||||
CG37   3841  TTCTGTGATTCCCCTCTATCGTGTTGCTTCATCAAGAACAATAATGGGTGCCCTCAAAAT  3900

CG104  3900  ATCGCAGCTTGTGGAATTTATAGCAATTGGTATCTTTATTGGAATATTAGGACTGAAAAT  3959
             ||||||||||||||||||||||||||||||||||||||||||||||||||||||||||||
CG37   3901  ATCGCAGCTTGTGGAATTTATAGCAATTGGTATCTTTATTGGAATATTAGGACTGAAAAT  3960

CG104  3960  TATATTTGTTGTAGAGATGATTTTTGGTAACAGTGATTGGGTAGTTAACTTGAGGTGGAA  4019
             ||||||||||||||||||||||||||||||||||||||||||||||||||||||||||||
CG37   3961  TATATTTGTTGTAGAGATGATTTTTGGTAACAGTGATTGGGTAGTTAACTTGAGGTGGAA  4020

CG104  4020  CATGGGGAGTGGTATGTCAATCCCATTTGTGGTTCTTCTTATTACTGCTTGTTCATCGTT  4079
             ||||||||||||||||||||||||||||||||||||||||||||||||||||||||||||
CG37   4021  CATGGGGAGTGGTATGTCAATCCCATTTGTGGTTCTTCTTATTACTGCTTGTTCATCGTT  4080

CG104  4080  TTGTCTGATGCTATGGTTGGCAGCTACCCCATTAAAATCTGCTACTACTATTGCCCAATT  4139
             ||||||||||||||||||||||||||||||||||||||||||||||||||||||||||||
CG37   4081  TTGTCTGATGCTATGGTTGGCAGCTACCCCATTAAAATCTGCTACTACTATTGCCCAATT  4140


CG104  4140  AGATGCTCAAGTATTGAACTGGGATATGGCAGAGGTTAGACCCGATTCATCTGAAGAGAG  4199
             ||||||||||||||||||||||||||||||||||||||||||||||||||||||||||||
CG37   4141  AGATGCTCAAGTATTGAACTGGGATATGGCAGAGGTTAGACCCGATTCATCTGAAGAGAG  4200

CG104  4200  GGAAAACATAGATTTGGGGAAAAGTTCATACAGTGCCGAGCCTATAGAAAGTCATTCTGA  4259
             ||||||||||||||||||||||||||||||||||||||||||||||||||||||||||||
CG37   4201  GGAAAACATAGATTTGGGGAAAAGTTCATACAGTGCCGAGCCTATAGAAAGTCATTCTGA  4260

CG104  4260  CCTATCTTCAACAAAGTTTGATTTTAATTTGCCTGAAAATATTATGGAACCTGATCAGGT  4319
             ||||||||||||||||||||||||||||||||||||||||||||||||||||||||||||
CG37   4261  CCTATCTTCAACAAAGTTTGATTTTAATTTGCCTGAAAATATTATGGAACCTGATCAGGT  4320

CG104  4320  TCTTGGTTCAGTTAATCAAAACGAGAATCGATCTAGTACTGTAGTTCCAAGCTCCCCAAA  4379
             ||||||||||||||||||||||||||||||||||||||||||||||||||||||||||||
CG37   4321  TCTTGGTTCAGTTAATCAAAACGAGAATCGATCTAGTACTGTAGTTCCAAGCTCCCCAAA  4380

CG104  4380  ATATGTACAAGAGGAACTTGAATCCACTGAGGAGTTAGTCTCATCCTCAATTGTGACTCA  4439
             ||||||||||||||||||||||||||||||||||||||||||||||||||||||||||||
CG37   4381  ATATGTACAAGAGGAACTTGAATCCACTGAGGAGTTAGTCTCATCCTCAATTGTGACTCA  4440

CG104  4440  CGATGTTCCTGATTCAACATTGGCTGACAAAAAGGTCTTAAAAATAGAGTCAGTGGAGGC  4499
             ||||||||||||||||||||||||||||||||||||||||||||||||||||||||||||
CG37   4441  CGATGTTCCTGATTCAACATTGGCTGACAAAAAGGTCTTAAAAATAGAGTCAGTGGAGGC  4500

CG104  4500  CGTTGAAAAGACTGTTGGACTCGATGGTGATTTGCGTTCTGAGAAGGATGATTATGAGGT  4559
             |||||||||||||||||||||||||||||||||.||||||||||||||||||||||||||
CG37   4501  CGTTGAAAAGACTGTTGGACTCGATGGTGATTTACGTTCTGAGAAGGATGATTATGAGGT  4560

CG104  4560  TGATAACTGGGAGGCTGAAGAGTCACTGAAAGAGATCTCTGGGAATATACCATCCTCAAC  4619
             ||||||||||||||||||||||||||||||||||||||||||||||||||||||||||||
CG37   4561  TGATAACTGGGAGGCTGAAGAGTCACTGAAAGAGATCTCTGGGAATATACCATCCTCAAC  4620

CG104  4620  ATCTGAGGGTCCTGGTTCTTTTAGAAGTATTGGTGGGAGAAGTGAAGAAGGTGGGAATGG  4679
             ||||||||||||||||||||||||||||||||||||||||||||||||||||||||||||
CG37   4621  ATCTGAGGGTCCTGGTTCTTTTAGAAGTATTGGTGGGAGAAGTGAAGAAGGTGGGAATGG  4680

CG104  4680  AACTGGTAGTCTTTCAAGGTTAGCTGGCCTCGGGCGTGCTGCAAGGCGCCAACTTACTGG  4739
             ||||||||||||||||||||||||||||||||||||||||||||||||||||||||||||
CG37   4681  AACTGGTAGTCTTTCAAGGTTAGCTGGCCTCGGGCGTGCTGCAAGGCGCCAACTTACTGG  4740
```

```
CG104  4740  AATTCTTGATGAATTTTGGGGACAATTGTATGATTTCCATGGGGTGCCTACTCAAGATGC  4799
             ||||||||||||||||||||||||||||||||||||||||||||||||||||||||||||
CG37   4741  AATTCTTGATGAATTTTGGGGACAATTGTATGATTTCCATGGGGTGCCTACTCAAGATGC  4800

CG104  4800  AAAGGTTAAGAAACTAGATTTGTTACTGGGTTTTACCTCTCTGAAATTGGATGCTGTTGG  4859
             ||||||||||||||||||||||||||||||||||||||||||||||||||||||||||||
CG37   4801  AAAGGTTAAGAAACTAGATTTGTTACTGGGTTTTACCTCTCTGAAATTGGATGCTGTTGG  4860

CG104  4860  TAAAGATTTTCCTCACTCATCACCTATTGGATGCAAAACATCCGATCCAATTTCTTCTAG  4919
             ||||||||||||||||||||||||||||||||||||||||||||||||||||||||||||
CG37   4861  TAAAGATTTTCCTCACTCATCACCTATTGGATGCAAAACATCCGATCCAATTTCTTCTAG  4920

CG104  4920  TTTGTACGACTCCCCCAAGAGTCAGAGGGTACAAAGTGGGTTAGAACCACCCTATGGGAT  4979
             ||||||||||||||||||||||||||||||||||||||||||||||||||||||||||||
CG37   4921  TTTGTACGACTCCCCCAAGAGTCAGAGGGTACAAAGTGGGTTAGAACCACCCTATGGGAT  4980

CG104  4980  ACAAAAGGGGCACCAGCCATTGTGGTCTAACCACATGCAGCATTGGGATGCATATGTGAA  5039
             ||||||||||||||||||||||||||||||||||||||||||||||||||||||||||||
CG37   4981  ACAAAAGGGGCACCAGCCATTGTGGTCTAACCACATGCAGCATTGGGATGCATATGTGAA  5040

CG104  5040  TAATTCTAGCCATAATGCTCTGGACTCTGGAGTGAAGCGATATTCTAGTTTGCGCAGTTT  5099
             ||||||||||||||||||||||||||||||||||||||||||||||||||||||||||||
CG37   5041  TAATTCTAGCCATAATGCTCTGGACTCTGGAGTGAAGCGATATTCTAGTTTGCGCAGTTT  5100

CG104  5100  GCCTTCTACTGAGAGTTGGGATTATCAGCCTGCCACAGTCCATGGCTATCAGTTAACTTA  5159
             ||||||||||||||||||||||||||||||||||||||||||||||||||||||||||||
CG37   5101  GCCTTCTACTGAGAGTTGGGATTATCAGCCTGCCACAGTCCATGGCTATCAGTTAACTTA  5160

CG104  5160  TCTGAGTAGAATGGCAAAGGACAGAAGTTCTGGTAATTCGAACGGTCAGTTGGATTCATC  5219
             ||||||||||||||||||||||||||||||||||||||.|||||||||||||||||||||
CG37   5161  TCTGAGTAGAATGGCAAAGGACAGAAGTTCTGGTAATTTGAACGGTCAGTTGGATTCATC  5220

CG104  5220  AGGCTCTAAATATCATACCTTGGGTGGTGGTGGTGCAGGCTTGCGAGACTCAGTTGCATT  5279
             ||||||||||||||||||||||||||||||||||||||||||||||||||||||||||||
CG37   5221  AGGCTCTAAATATCATACCTTGGGTGGTGGTGGTGCAGGCTTGCGAGACTCAGTTGCATT  5280

CG104  5280  TGCAATGGGGCAAAAGTTGCAAAATGGCTTGGGTGCTTGTCAGCAGGCGGCTCCCCCAGG  5339
             ||||||||||||||||||||||||||||||||||||||||||||||||||||||||||||
CG37   5281  TGCAATGGGGCAAAAGTTGCAAAATGGCTTGGGTGCTTGTCAGCAGGCGGCTCCCCCAGG  5340

CG104  5340  TTTTTCCAACATCACAGTATCCAGGAAACCTTCTTCCGAATCTGAAAGGAAATATTATGA  5399
             ||||||||||||||||||||||||||||||||||||||||||||||||||||||||||||
CG37   5341  TTTTTCCAACATCACAGTATCCAGGAAACCTTCTTCCGAATCTGAAAGGAAATATTATGA  5400

CG104  5400  TCATTCTCTTCTGGAACTGGTGAGAATTTAGTGAGTGTATCTAACACAAAGAAATACCA  5459
             ||||||||.||||||||||||||||||||||||||||||||||||||||||||||||||
CG37   5401  TCATTCTCCTTCTGGAACTGGTGAGAATTTAGTGAGTGTATCTAACACAAAGAAATACCA  5460

CG104  5460  TAGCTTACCGGATATTCACCGTGATCAGCACACATCAGATAAGAGTTCTCAGTGGGATAA  5519
             ||||||||||||||||||||||||||||||||||||||||||||||||||||||||||||
CG37   5461  TAGCTTACCGGATATTCACCGTGATCAGCACACATCAGATAAGAGTTCTCAGTGGGATAA  5520

CG104  5520  TGTGAGTGGTTATGGAACATCTATTGGTAGAATAACAGCTCGTGGAGTGTCCACAAATTC  5579
             ||||||||||||||||||||||||||||||||||||||||||||||||||||||||||||
CG37   5521  TGTGAGTGGTTATGGAACATCTATTGGTAGAATAACAGCTCGTGGAGTGTCCACAAATTC  5580

CG104  5580  TGGATCAAGATTAGTTTCTCCTTTAGCATTTGATGAACTATCTCCTGCAAATGTCTACAG  5639
             ||||||||||||||||||||||||||||||||||||||||||||||||||||||||||||
CG37   5581  TGGATCAAGATTAGTTTCTCCTTTAGCATTTGATGAACTATCTCCTGCAAATGTCTACAG  5640

CG104  5640  TGGTGCATTATCACCACAAATGAATCCTCATCTGGATTCTGGATCTTTCTGGCATAGACA  5699
             ||||||||||||||||||||||||||||||||||||||||||||||||||||||||||||
CG37   5641  TGGTGCATTATCACCACAAATGAATCCTCATCTGGATTCTGGATCTTTCTGGCATAGACA  5700

CG104  5700  GCCTTCTGAGCAATTTGGCTTGGACAAAAATAGCAACTCCGAGAGTAAAGGAATTGGGAG  5759
             ||||||||||||||||||||||||||||||||||||||||||||||||||||||||||||
```

```
CG37    5701  GCCTTCTGAGCAATTTGGCTTGGACAAAAATAGCAACTCCGAGAGTAAAGGAATTGGGAG  5760

CG104   5760  GCTGCATTCAATTAGTCACGAAGCTTCTTTTGTTGTTAATTCAGAGGCCAGGCTTCTCCA  5819
              ||||||||||||||||||||||||||||||||||||||||||||||||||||||||||||
CG37    5761  GCTGCATTCAATTAGTCACGAAGCTTCTTTTGTTGTTAATTCAGAGGCCAGGCTTCTCCA  5820

CG104   5820  GTCCTTCAGAGACTGCATTGTCAAACTTCTGAAATTAGAAGGATCAGACTGGTTATTTGG  5879
              ||||||||||||||||||||||||||||||||||||||||||||||||||||||||||||
CG37    5821  GTCCTTCAGAGACTGCATTGTCAAACTTCTGAAATTAGAAGGATCAGACTGGTTATTTGG  5880

CG104   5880  GCAAAGTGATGGTGCTGACGAGGAGCTAATTGATTGTGTAGCTGCCAGGGAGAAATTTCT  5939
              ||||||||||||||||||||||||.|||||||||||||||||||||||||||||||||||
CG37    5881  GCAAAGTGATGGTGCTGACGAGGAACTAATTGATTGTGTAGCTGCCAGGGAGAAATTTCT  5940

CG104   5940  TTATGAAGCTGAGGCAAGGGAGATGGGTCGGGTGGTCCGCATGAAAGAATCTCCTTCATT  5999
              ||||||||||||||||||||||||||||||||||||||||||||||||||.|||||||||
CG37    5941  TTATGAAGCTGAGGCAAGGGAGATGGGTCGGGTGGTCCGCATGAAAGAATGTCCTTCATT  6000

CG104   6000  TTCTCCTGATAGGAGACCAGGTTCTGGAATGAAGAATGATACAAATTTCTCCAATGTTTC  6059
              ||||||||||||||||||||||||||||||||||||||||||||||||||||||||||||
CG37    6001  TTCTCCTGATAGGAGACCAGGTTCTGGAATGAAGAATGATACAAATTTCTCCAATGTTTC  6060

CG104   6060  TATTTCCTCTGTACCTCATTGTGGAGAAGGCTGTATTTGGAGATCAGATTTGATTGTAAG  6119
              ||||||||||||||||||||||||||||||||||||||||||||||||||||||||||||
CG37    6061  TATTTCCTCTGTACCTCATTGTGGAGAAGGCTGTATTTGGAGATCAGATTTGATTGTAAG  6120

CG104   6120  TTTTGGTGTATGGTGCATTCACCGTATTCTAGATCTCTCACTTATGGAAAGTCGGCCTGA  6179
              ||||||||||||||||||||||||||||||||||||||||||||||||||||||||||||
CG37    6121  TTTTGGTGTATGGTGCATTCACCGTATTCTAGATCTCTCACTTATGGAAAGTCGGCCTGA  6180


CG104   6180  ACTATGGGGAAAATATACCTATGTACTCAATCGTCTTCAGGTAAACATTGTCTGTTTGTC  6239
              ||||||||||||||||||||||||||||||||||||||||||||||||||||||||||||
CG37    6181  ACTATGGGGAAAATATACCTATGTACTCAATCGTCTTCAGGTAAACATTGTCTGTTTGTC  6240

CG104   6240  TTTGTATCATTAGCATATAACCTAGTGTATCAGGTAAAAGTCTCTTACTGAAAATACCAT  6299
              ||||||||||||||||||||||||||||||||||||||||||||||||||||||||||||
CG37    6241  TTTGTATCATTAGCATATAACCTAGTGTATCAGGTAAAAGTCTCTTACTGAAAATACCAT  6300

CG104   6300  TGGTCTTTGTTGAGATCACATCCATTTACATAAGTTGAGAGTTCGATCAGTTGCAGGGCT  6359
              |||||.||||||||||||||||||||||||||||||||||||||||||||||||||||||
CG37    6301  TGGTCGTTGTTGAGATCACATCCATTTACATAAGTTGAGAGTTCGATCAGTTGCAGGGCT  6360

CG104   6360  GACATATTTTGCAGTCAAAATGGCT---AGTAGTTTTGGATTATATGAATTACAGATATT  6416
              |||||||||||||||||||||||||   ||||||||||||||||||||||||||||||||
CG37    6361  GACATATTTTGCAGTCAAAATGGCTAGAAGTAGTTTTGGATTATATGAATTACAGATATT  6420

CG104   6417  CTAATGGGATCAATTGTTTTATGCATCCGACCTAAGTGTTTGGGTTGAGAGTTTGTATTG  6476
              ||||||||||||||||||||||||||||||||||||||||||||||||||||||||||||
CG37    6421  CTAATGGGATCAATTGTTTTATGCATCCGACCTAAGTGTTTGGGTTGAGAGTTTGTATTG  6480

CG104   6477  TTTGCATACGGATATTTTCAGAATATGTTGATTTTAATCTCTTCTGCCCTGAGTGTTTGG  6536
              ||||||||||||| ||||||||||||||||||||||||||||||||||||||||||||||
CG37    6481  TTTGCATACGGATGTTTTCAGAATATGTTGATTTTAATCTCTTCTGCCCTGAGTGTTTGG  6540

CG104   6537  GTGGTTTTGATAATGTGCTTACTGGCTCCATTTAGAGTCAATATGAAAGATACAAATAGT  6596
              ||||||||||||||||||||||||||||||||||||||||||||||||| ||||||||||
CG37    6541  GTGGTTTTGATAATGTGCTTACTGGCTCCATTTAGAGTCAATATGAAAGCTACAAATAGT  6600

CG104   6597  AAGGAACATGCTTTTCCATCTTAAGATAATATGCACAACTTGTATGTGCATGTCGTAGGA  6656
              ||||||||||||||||||||||||||||||||||||||||||||||||||||||||||||
CG37    6601  AAGGAACATGCTTTTCCATCTTAAGATAATATGCACAACTTGTATGTGCATGTCGTAGGA  6660

CG104   6657  ATTGTAATCAAATTTTCCTTTTAGATCGTCATGGAAGATTGTGAAATGAGGGTGTACTGT  6716
              ||||||||||||||||||||||||||||||||||||||||||||||||||||||||||||
CG37    6661  ATTGTAATCAAATTTTCCTTTTAGATCGTCATGGAAGATTGTGAAATGAGGGTGTACTGT  6720

CG104   6717  CCACTGATTTGCTTTTACTCCTTGTGAACTCTAATGGCAAACATTCTTTCAGGGTATTAT  6776
```

```
              ||||||||||||||||||||||||||||||||||||||||||||||||||||||||||||
CG37    6721  CCACTGATTTGCTTTTACTCCTTGTGAACTCTAATGGCAAACATTCTTTCAGGGTATTAT  6780

CG104   6777  CGATCCTGCATTTTCGAAGCCTCGTATACCGATGCCACCATGCTTCTGCCTCCAAATTCC  6836
              ||||||||||||||||||||||||||||||||||||||||||||||||||||||||||||
CG37    6781  CGATCCTGCATTTTCGAAGCCTCGTATACCGATGCCACCATGCTTCTGCCTCCAAATTCC  6840

CG104   6837  CCAAGCATTCCAGCAGAGGTCAAGCCCACAAATTGCAAATGGAATGTTGCCTCCTGCTGC  6896
              ||||||||||||||||||||||||||||||||||||||||||||||||||||||||||||
CG37    6841  CCAAGCATTCCAGCAGAGGTCAAGCCCACAAATTGCAAATGGAATGTTGCCTCCTGCTGC  6900

CG104   6897  AAAACCTGGCAAGGGAAAATGCACCACTGCTGCAATGCTTCTGGATATGGTCAAGGATGT  6956
              ||||||||||||||||||||||||||||||||||||||||||||||||||||||||||||
CG37    6901  AAAACCTGGCAAGGGAAAATGCACCACTGCTGCAATGCTTCTGGATATGGTCAAGGATGT  6960

CG104   6957  GGAGATAGCCATCTCTTGCCGAAAAGGTCGAACTGGTACAGCAGCCGGCGACGTAGCTTT  7016
              ||||||||||||||||||||||||||||||||||||||||||||||||||||||||||||
CG37    6961  GGAGATAGCCATCTCTTGCCGAAAAGGTCGAACTGGTACAGCAGCCGGCGACGTAGCTTT  7020

CG104   7017  CCCAAAGGGGAAGGAGAACTTGGCTTCAGTCCTCAAACGCTACAAGCGCCGATTATCCAA  7076
              ||||||||||||||||||||||||||||||||||||||||||||||||||||||||||||
CG37    7021  CCCAAAGGGGAAGGAGAACTTGGCTTCAGTCCTCAAACGCTACAAGCGCCGATTATCCAA  7080

CG104   7077  TAAACCAGTTGCCACTCACGAAGTATCATCTATTTCACGCAAGATTTCAGCAACATCCGT  7136
              ||||||||||||||||||||||||||||||||||||||||||||||||||||||||||||
CG37    7081  TAAACCAGTTGCCACTCACGAAGTATCATCTATTTCACGCAAGATTTCAGCAACATCCGT  7140

CG104   7137  TCCTTATAGCTCATAGTATTTACCCAAAAATGGTGATCAAATCACCCAGCTGTTTAATTT  7196
              ||||||||||||||||||||||||||||||||||||||| ||||||||||||||||||||
CG37    7141  TCCTTATAGCTCATAGTATTTACCCAAAAATGGTGATCAGATCACCCAGCTGTTTAATTT  7200


CG104   7197  TGGAAAGCAGCTCATGGTTTGGAACGAGATGCCCTCGTCTTGGTCTTTACTCTCTCTCAA  7256
              ||||||||||||||||||||||||||||||||||||||||||||||||||||||||||||
CG37    7201  TGGAAAGCAGCTCATGGTTTGGAACGAGATGCCCTCGTCTTGGTCTTTACTCTCTCTCAA  7260

CG104   7257  AACATGTATCAAGGCTCTTTGCTGCGAATTTTCTTCTCACATGTTAAAATATGATTAGGA  7316
              ||||||||||||||||||||||||||||||||||||||||||||||||||||||||||||
CG37    7261  AACATGTATCAAGGCTCTTTGCTGCGAATTTTCTTCTCACATGTTAAAATATGATTAGGA  7320

CG104   7317  TGTGACTCAATGGACCCTTAGTTGCAGCAAAACTCAGGAAACTGGTGCAAACACACATGG  7376
              |||||||||||||||||||||||||||||||||||||||||||||||||||||| |||||
CG37    7321  TGTGACTCAATGGACCCTTAGTTGCAGCAAAACTCAGGAAACTGGTGCAAACACGCATGG  7380

CG104   7377  AAAGTTGATTGTTATGATGGTTGCACTACTTACTTGTATCAAAGTAATTGCTGGAGGAGA  7436
              ||||||||||||||||||||||||||||||||||||||||||||||||||||||||||||
CG37    7381  AAAGTTGATTGTTATGATGGTTGCACTACTTACTTGTATCAAAGTAATTGCTGGAGGAGA  7440

CG104   7437  GATCACAAGGTGACTTTGAAAAGTTGAAAAAAAAATGTTAGTATATAGCAGACAGAGCTA  7496
              ||||||||||||||||||||||||||||||||||||||||||||||||||||||||||||
CG37    7441  GATCACAAGGTGACTTTGAAAAGTTGAAAAAAAAATGTTAGTATATAGCAGACAGAGCTA  7500

CG104   7497  ATGTGCTGTATATATTGTCTGTGCAAAACTTTCTGAAACATTTCAAATCTCTTTCCACAA  7556
              ||||||||||||||||||||||||||||||||||||||||||||||||||||||||||||
CG37    7501  ATGTGCTGTATATATTGTCTGTGCAAAACTTTCTGAAACATTTCAAATCTCTTTCCACAA  7560

CG104   7557  AAGCTATTCTAGATCTTGCTGTTTGTTGTTAGATCATGCAGAAACTTTCTTCTGTTGCCC  7616
              ||||||||||||||||||||||||||||||||||||||||||||||||||||||||||||
CG37    7561  AAGCTATTCTAGATCTTGCTGTTTGTTGTTAGATCATGCAGAAACTTTCTTCTGTTGCCC  7620

CG104   7617  GTTTCGGTGAGTCAAAAGATAACTACATACTCTAGGG  7653
              |||||||||||||||||||||||||||||||||||||
CG37    7621  GTTTCGGTGAGTCAAAAGATAACTACATACTCTAGGG  7657
```

Supplementary Figure S3. DNA sequence alignments of the candidate gene, *Csa6G445210* and *Csa6G445230*, between CG104 and CG37 (the highlight indicates the exons)

Csa6G445210

```
CG104  1    MITFMDSKEKLKEVEKCLDPQLWHACAGGMVQMPPVNARVFYFPQGHAEHSCAPVDFRNC  60
            ||||||||||||||||||||||||||||||||||||||||||||||||||||||||||||
CG37   1    MITFMDSKEKLKEVEKCLDPQLWHACAGGMVQMPPVNARVFYFPQGHAEHSCAPVDFRNC  60

CG104  61   PKVPSYTLCRVSAIKFLADPDTDEVFAKLRLIPINGSELDFEDDGIGRLNGSEQDKPTSF  120
            ||||||||||||||||||||||||||||||||||||||||||||||||||||||||||||
CG37   61   PKVPSYTLCRVSAIKFLADPDTDEVFAKLRLIPINGSELDFEDDGIGRLNGSEQDKPTSF  120

CG104  121  AKTLTQSDANNGGGFSVPRYCAETIFPRLDYSADPPVQTILAKDVHGETWKFRHIYRGTP  180
            ||||||||||||||||||||||||||||||||||||||||||||||||||||||||||||
CG37   121  AKTLTQSDANNGGGFSVPRYCAETIFPRLDYSADPPVQTILAKDVHGETWKFRHIYRGTP  180

CG104  181  RRHLLTTGWSTFVNHKKLVAGDSIVFLRAENGDLCVGIRRAKRGIGDGPESSCGWNPAGG  240
            ||||||||||||||||||||||||||||||||||||||||||||||||||||||||||||
CG37   181  RRHLLTTGWSTFVNHKKLVAGDSIVFLRAENGDLCVGIRRAKRGIGDGPESSCGWNPAGG  240

CG104  241  NCAVSYGAFSAFLREDDNRLTRSANGMNGNGSLMGKGKVKPESVTEAAKLASNGQPFEII  300
            ||||||||||||||||||||||||||||||||||||||||||||||||||||||||||||
CG37   241  NCAVSYGAFSAFLREDDNRLTRSANGMNGNGSLMGKGKVKPESVTEAAKLASNGQPFEII  300

CG104  301  FYPRASTPEFCVKAALVKAALQIRWCSGMRFKMAFETEDSSRISWFMGTINSVQVSDPLR  360
            ||||||||||||||||||||||||||||||||||||||||||||||||||||||||||||
CG37   301  FYPRASTPEFCVKAALVKAALQIRWCSGMRFKMAFETEDSSRISWFMGTINSVQVSDPLR  360

CG104  361  WPESPWRLLQVTWDEPDLLQNVKRVSPWLVELVSSMSPIHLAPFSPPRKKFRYPQHPDFP  420
            ||||||||||||||||||||||||||||||||||||||||||||||||||||||||||||
CG37   361  WPESPWRLLQVTWDEPDLLQNVKRVSPWLVELVSSMSPIHLAPFSPPRKKFRYPQHPDFP  420

CG104  421  LDNQPPVPSFSSYLHGTGSPFGCPPDNNPAGMQGARHAHFGLSLSDFHVSKLQSGLFSIG  480
            ||||||||||||||||||||||||||||||||||||||||||||||||||||||||||||
CG37   421  LDNQPPVPSFSSYLHGTGSPFGCPPDNNPAGMQGARHAHFGLSLSDFHVSKLQSGLFSIG  480

CG104  481  YRSLDPAAGSTRLSGNVMTEKPSMSENVSCLLTMAHSTQASKKFDGVKTPQLILFGRPIL  540
            ||||||||||||||||||||||||||||||||||||||||||||||||||||||||||||
CG37   481  YRSLDPAAGSTRLSGNVMTEKPSMSENVSCLLTMAHSTQASKKFDGVKTPQLILFGRPIL  540

CG104  541  TELQMSQSFSGDTVSPVGTGNSSSDGNGDKMTNLSDGSGSALHQQGLPEGSAGENFQWYK  600
            ||||||||||||||||||||||||||||||||||||||||||||||||||||||||||||
CG37   541  TELQMSQSFSGDTVSPVGTGNSSSDGNGDKMTNLSDGSGSALHQQGLPEGSAGENFQWYK  600

CG104  601  DNCQEIDPNLDIGHCKVFMESEDVGRTLDLSSLGSYEELYRKLGNMFGIDNSETLNHVLY  660
            || |||||||||||||||||||||||||||||||||||||||||||||||||||||||||
CG37   601  DNRQEIDPNLDIGHCKVFMESEDVGRTLDLSSLGSYEELYRKLGNMFGIDNSETLNHVLY  660

CG104  661  RDVSGAVKHVGDEQFSDFIKTARRLTILTDSGSNNVGA  698
            ||||||||||||||||||||||||||||||||||||||
CG37   661  RDVSGAVKHVGDEQFSDFIKTARRLTILTDSGSNNVGA  698
```

Csa6G445230

```
CG104  1    MESTTLHTTHQSGAIHRFIPFIAPALLVSISYVDPGKWAATVEGGARFGFDLFVLVLLFN  60
            ||||||||||||||||||||||||||||||||||||||||||||||||||||||||||||
CG37   1    MESTTLHTTHQSGAIHRFIPFIAPALLVSISYVDPGKWAATVEGGARFGFDLFVLVLLFN  60

CG104  61   LAAILCQYLSASIGVVTGRGLAQICNEEYDKCTCFFLGIQAEASVILLDLNMILGISNGL  120
            ||||||||||||||||||||||||| ||||||||||||||||||||||||||||||||||
CG37   61   LAAILCQYLSASIGVVTGRGLAQICSEEYDKCTCFFLGIQAEASVILLDLNMILGISNGL  120

CG104  121  NLLLGWDLFTCVLLTGVAAALFPPFADLLEDGRAKFLYICMAGFVLLSLVLGVLISQPEI  180
            ||||||||||||||||||||||||||||||||||||||||||||||||||||||||||||
CG37   121  NLLLGWDLFTCVLLTGVAAALFPPFADLLEDGRAKFLYICMAGFVLLSLVLGVLISQPEI  180

CG104  181  PLSMNLMPTRLNGESAFTLMSLLGASVMPHNFYVHSSIVQQHQSPPNISKEVSCYNHLFA  240
            ||||||||||||||||||||||||||||||||||||||||||||||||||||||||||||
CG37   181  PLSMNLMPTRLNGESAFTLMSLLGASVMPHNFYVHSSIVQQHQSPPNISKEVSCYNHLFA  240
```

```
CG104  241   IFCIFSGIYVVNNVLMNSAANVFYSSGLALHTFTDALSLMEQVFGSSVVYVLFLLVLFLS  300
             |||||||||||||||||||||||||||||||||:||||||||||||||||||||||||||
CG37   241   IFCIFSGIYVVNNVLMNSAANVFYSSGLALHTFSDALSLMEQVFGSSVVYVLFLLVLFLS  300

CG104  301   NQITALTWSLGGQLVLTNFLKLDIPGWLHCATIRIIAIIPALCCVWSSGAEGMYQLLIFS  360
             ||||||||||||||||||||||||||||||||||||||||||||||||||||||||||||
CG37   301   NQITALTWSLGGQLVLTNFLKLDIPGWLHCATIRIIAIIPALCCVWSSGAEGMYQLLIFS  360

CG104  361   QVMVALLLPSSVIPLYRVASSRTIMGALKISQLVEFIAIGIFIGILGLKIIFVVEMIFGN  420
             ||||||||||||||||||||||||||||||||||||||||||||||||||||||||||||
CG37   361   QVMVALLLPSSVIPLYRVASSRTIMGALKISQLVEFIAIGIFIGILGLKIIFVVEMIFGN  420

CG104  421   SDWVVNLRWNMGSGMSIPFVVLLITACSSFCLMLWLAATPLKSATTIAQLDAQVLNWDMA  480
             ||||||||||||||||||||||||||||||||||||||||||||||||||||||||||||
CG37   421   SDWVVNLRWNMGSGMSIPFVVLLITACSSFCLMLWLAATPLKSATTIAQLDAQVLNWDMA  480

CG104  481   EVRPDSSEERENIDLGKSSYSAEPIESHSDLSSTKFDFNLPENIMEPDQVLGSVNQNENR  540
             ||||||||||||||||||||||||||||||||||||||||||||||||||||||||||||
CG37   481   EVRPDSSEERENIDLGKSSYSAEPIESHSDLSSTKFDFNLPENIMEPDQVLGSVNQNENR  540

CG104  541   SSTVVPSSPKYVQEELESTEELVSSSIVTHDVPDSTLADKKVLKIESVEAVEKTVGLDGD  600
             ||||||||||||||||||||||||||||||||||||||||||||||||||||||||||||
CG37   541   SSTVVPSSPKYVQEELESTEELVSSSIVTHDVPDSTLADKKVLKIESVEAVEKTVGLDGD  600

CG104  601   LRSEKDDYEVDNWEAEESLKEISGNIPSSTSEGPGSFRSIGGRSEEGGNGTGSLSRLAGL  660
             ||||||||||||||||||||||||||||||||||||||||||||||||||||||||||||
CG37   601   LRSEKDDYEVDNWEAEESLKEISGNIPSSTSEGPGSFRSIGGRSEEGGNGTGSLSRLAGL  660

CG104  661   GRAARRQLTGILDEFWGQLYDFHGVPTQDAKVKKLDLLLGFTSLKLDAVGKDFPHSSPIG  720
             ||||||||||||||||||||||||||||||||||||||||||||||||||||||||||||
CG37   661   GRAARRQLTGILDEFWGQLYDFHGVPTQDAKVKKLDLLLGFTSLKLDAVGKDFPHSSPIG  720

CG104  721   CKTSDPISSSLYDSPKSQRVQSGLEPPYGIQKGHQPLWSNHMQHWDAYVNNSSHNALDSG  780
             ||||||||||||||||||||||||||||||||||||||||||||||||||||||||||||
CG37   721   CKTSDPISSSLYDSPKSQRVQSGLEPPYGIQKGHQPLWSNHMQHWDAYVNNSSHNALDSG  780

CG104  781   VKRYSSLRSLPSTESWDYQPATVHGYQLTYLSRMAKDRSSGNSNGQLDSSGSKYHTLGGG  840
             |||||||||||||||||||||||||||||||||||||||||| |||||||||||||||||
CG37   781   VKRYSSLRSLPSTESWDYQPATVHGYQLTYLSRMAKDRSSGNLNGQLDSSGSKYHTLGGG  840

CG104  841   GAGLRDSVAFAMGQKLQNGLGACQQAAPPGFSNITVSRKPSSESERKYYDHSLSGTGENL  900
             |||||||||||||||||||||||||||||||||||||||||||||||||||| |||||||
CG37   841   GAGLRDSVAFAMGQKLQNGLGACQQAAPPGFSNITVSRKPSSESERKYYDHSPSGTGENL  900

CG104  901   VSVSNTKKYHSLPDIHRDQHTSDKSSQWDNVSGYGTSIGRITARGVSTNSGSRLVSPLAF  960
             ||||||||||||||||||||||||||||||||||||||||||||||||||||||||||||
CG37   901   VSVSNTKKYHSLPDIHRDQHTSDKSSQWDNVSGYGTSIGRITARGVSTNSGSRLVSPLAF  960

CG104  961   DELSPANVYSGALSPQMNPHLDSGSFWHRQPSEQFGLDKNSNSESKGIGRLHSISHEASF  1020
             ||||||||||||||||||||||||||||||||||||||||||||||||||||||||||||
CG37   961   DELSPANVYSGALSPQMNPHLDSGSFWHRQPSEQFGLDKNSNSESKGIGRLHSISHEASF  1020

CG104  1021  VVNSEARLLQSFRDCIVKLLKLEGSDWLFGQSDGADEELIDCVAAREKFLYEAEAREMGR  1080
             ||||||||||||||||||||||||||||||||||||||||||||||||||||||||||||
CG37   1021  VVNSEARLLQSFRDCIVKLLKLEGSDWLFGQSDGADEELIDCVAAREKFLYEAEAREMGR  1080

CG104  1081  VVRMKESPSFSPDRRPGSGMKNDTNFSNVSISSVPHCGEGCIWRSDLIVSFGVWCIHRIL  1140
             ||||||.|||||||||||||||||||||||||||||||||||||||||||||||||||||
CG37   1081  VVRMKECPSFSPDRRPGSGMKNDTNFSNVSISSVPHCGEGCIWRSDLIVSFGVWCIHRIL  1140

CG104  1141  DLSLMESRPELWGKYTYVLNRLQGIIDPAFSKPRIPMPPCFCLQIPQAFQQRSSPQIANG  1200
             ||||||||||||||||||||||||||||||||||||||||||||||||||||||||||||
CG37   1141  DLSLMESRPELWGKYTYVLNRLQGIIDPAFSKPRIPMPPCFCLQIPQAFQQRSSPQIANG  1200

CG104  1201  MLPPAAKPGKGKCTTAAMLLDMVKDVEIAISCRKGRTGTAAGDVAFPKGKENLASVLKRY  1260
             ||||||||||||||||||||||||||||||||||||||||||||||||||||||||||||
CG37   1201  MLPPAAKPGKGKCTTAAMLLDMVKDVEIAISCRKGRTGTAAGDVAFPKGKENLASVLKRY  1260
```

```
CG104  1261  KRRLSNKPVATHEVSSISRKISATSVPYSS  1290
             ||||||||||||||||||||||||||||||
CG37   1261  KRRLSNKPVATHEVSSISRKISATSVPYSS  1290
```

Supplementary Figure S4. Amino acid sequence alignments of the candidate genes, *Csa6G445210* and *Csa6G445230*, between CG104 and CG37
